# Transcriptional variation of sensory-related genes in natural populations of *Aedes albopictus*

**DOI:** 10.1186/s12864-020-06956-6

**Published:** 2020-08-07

**Authors:** Ludvik M. Gomulski, Mosè Manni, Davide Carraretto, Tony Nolan, Daniel Lawson, José M. Ribeiro, Anna R. Malacrida, Giuliano Gasperi

**Affiliations:** 1 Department of Biology and Biotechnology, University of Pavia, Pavia, Italy; 2 Department of Genetic Medicine and Development, University of Geneva Medical School, and Swiss Institute of Bioinformatics, Geneva, Switzerland; 3 Department of Vector Biology, Liverpool School of Tropical Medicine, Liverpool, UK; 4 Department of Life Sciences, Imperial College London, London, UK; 5 NIAID, Laboratory of Malaria and Vector Research, NIH, Rockville, MD 20852 USA

**Keywords:** Asian tiger mosquito, Invasive species, Differential transcription, Single nucleotide polymorphisms, Chemoreception

## Abstract

**Background:**

The Asian tiger mosquito, *Aedes albopictus*, is a highly dangerous invasive vector of numerous medically important arboviruses including dengue, chikungunya and Zika. In four decades it has spread from tropical Southeast Asia to many parts of the world in both tropical and temperate climes. The rapid invasion process of this mosquito is supported by its high ecological and genetic plasticity across different life history traits. Our aim was to investigate whether wild populations, both native and adventive, also display transcriptional genetic variability for functions that may impact their biology, behaviour and ability to transmit arboviruses, such as sensory perception.

**Results:**

Antennal transcriptome data were derived from mosquitoes from a native population from Ban Rai, Thailand and from three adventive Mediterranean populations: Athens, Greece and Arco and Trento from Italy. Clear inter-population differential transcriptional activity was observed in different gene categories related to sound perception, olfaction and viral infection. The greatest differences were detected between the native Thai and the Mediterranean populations. The two Italian populations were the most similar.

Nearly one million quality filtered SNP loci were identified.

**Conclusion:**

The ability to express this great inter-population transcriptional variability highlights, at the functional level, the remarkable genetic flexibility of this mosquito species. We can hypothesize that the differential expression of genes, including those involved in sensory perception, in different populations may enable *Ae. albopictus* to exploit different environments and hosts, thus contributing to its status as a global vector of arboviruses of public health importance.

The large number of SNP loci present in these transcripts represents a useful addition to the arsenal of high-resolution molecular markers and a resource that can be used to detect selective pressure and adaptive changes that may have occurred during the colonization process.

## Background

The Asian tiger mosquito, *Aedes albopictus*, is one of the most invasive species in the world and is increasingly becoming an important vector of virus-induced diseases (chikungunya, dengue, Zika) [1]. From its home-range in tropical Southeast Asia, where it was a zoophilic forest species [2], it spread initially to the Indian and Pacific islands [3] and then over the last 40 years, it rapidly spread to numerous tropical, subtropical and temperate regions in Europe, the Americas and Africa [4–7]. It is able to tolerate climate/environment interactions that differ from those of its home range [8–10]. Its ability to readily adapt to new habitats is associated with numerous environmentally-induced adaptive biological traits including eggs that can diapause and survive in cooler, less favourable conditions [2, 4, 10, 11] and opportunistic feeding behaviour on a wide range of animals [12]. Human activities also create new breeding and trophic niches of adaptation in the vicinity of dwellings, strengthening their association with humans [13, 14]. The threat of viral transmission has led to the establishment of surveillance and vector control strategies that aim to minimize the impact on public health. Successful intervention and control gains from knowledge of the genetic, ecological and behavioural traits of mosquito populations [15], as well as the history and dynamics of invasive processes. Numerous studies have attempted to unravel different aspects of the *Ae. albopictus* invasion process and to infer the genetic relationships between populations at the micro- and macro-geographic levels [10, 14, 16–23]. From these studies it appears that the global invasion process was aided by independent trans- and inter-continental introductions that may have facilitated the rapid establishment of adventive populations through admixture of unrelated genomes. As a result, a great deal of intra-population variability has been detected in both Southeast Asian populations and in the globally distributed adventive populations [19]. We have found that this genetic variability extends to the genetic mechanisms controlling vector competence for the chikungunya virus [19]. Here we used a comparative approach to verify whether this high genetic variability is also present at the transcriptional level.

We chose to sample the antennal transcriptomes of *Ae. albopictus* as the antennae are highly sophisticated peripheral sensory structures that act as intermediates between the mosquito, its environment and hosts. The antennae are implicated in the sensory reception of stimuli such as sound, heat, and odours [24] that are of vital importance to mosquitoes for mate location, the detection of suitable blood meal hosts, nectar sources, resting and oviposition sites [25]. Given the importance of the antennae in interactions of the mosquito with blood meal hosts, they will also influence disease transmission [26–28]. Moreover, the analysis of genetic diversity in sensory perception may help to improve the efficiency of control interventions [29, 30].

On this basis, we compared the antennal transcriptomes of wild *Ae. albopictus* samples collected from a population in the home range, Thailand, and populations from the Mediterranean invasion area with the aim of investigating the presence of transcriptional variation for genes controlling sensory perception functions. We extended our analyses to determine the presence and nature of single nucleotide polymorphisms (SNPs) in the transcripts present in the considered populations. We discovered the presence of a high level of inter-population transcriptional and SNP variability in sensory related genes. High levels of variability were detected in genes associated with sound, vision and odorant perception, nitrogen assimilation, and flight behaviour. We also identified variability related to viral replication in the populations. The ability to express this extensive inter-population genetic variability highlights the remarkable genetic flexibility of this mosquito species, that invaded much of the world in four decades, while its counterpart, *Aedes aegypti*, did so in four centuries [5].

## Methods

### Mosquito samples

Eggs were collected from three eco-geographic areas: one from the native range in Southeast Asia, Thailand, Ban Rai, Lampang Province (18°18′36″N 99°33′00″E, three collections, May–June 2012) and three from the Mediterranean area, Greece, Athens (37°57′52″N 23°43′44″E, one collection, September 2011) and northern Italy, Arco (45°55′42″N 10°56′02″E, two collections, October 2011) and Trento (46°04′12″N 11°08′17″E, two collections, September 2011). The Ban Rai population derives from a tropical savanna climate (Köppen climate classification) with a mean summer temperature of around 35 °C. Monthly rainfall during the collection period averaged 140 mm. The area is a mixture of woodland and agricultural land with mainly rice cultivation. The Athens population derives from a temperate, highly urbanised setting with high levels of anthropogenic environmental transformation (anthropization) and pollution [31]. The average temperature during the collection period was 25 °C with a monthly precipitation of 23 mm. The Trentino/Arco populations are from a mostly mountainous and forested area with a temperate climate, and a lower extent of human intervention with limited agricultural use. Average monthly temperature during the collection period was 19.9 °C and 91 mm rainfall for Trento and 13.4 °C and 100 mm rainfall for Arco. For each locality, the eggs were collected and pooled from several oviposition traps to minimize inbreeding effects. The eggs were brought into the insectary maintained at 26 °C with 70% relative humidity and a 12:12 h (light: dark) photoperiod and were hatched in autoclaved water and fed on fish food pellets (Tetra) daily as necessary. Pupae were collected in plastic cups containing 50 ml water and placed in 20 cm cube cages from which newly emerged adults were collected each morning. Both sexes were maintained together in the same cage with 20% sugar solution at the same temperature/humidity/photoperiod as previously described. At 2 to 3 days of age the antennae were removed from equal numbers of G0 male and female individuals from each population sample and placed immediately in 200 μl of chilled Trizol (Invitrogen). The number of antennae pairs collected per population was 420 for Arco, 435 for Athens, 764 for Ban Rai and 921 for Trento.

### RNA sample preparation and sequencing

For each population collection, total RNA was extracted from the antennae using Trizol (Invitrogen), followed by treatment with DNase (DNAfree, Ambion). The RNA sample from each collection was further purified using Micro Bio-Spin P-30 gel columns (BioRad). After quantification using an Agilent 2100 Bioanalyser the RNAs from the different collections in each of the four sampling sides were pooled. mRNA isolation and cDNA library preparation for each of the four samples were performed using the Illumina TruSeq RNA sample preparation kit (Illumina Inc., San Diego, CA, USA). The libraries were barcoded, pooled and sequenced as a 100 bp paired end run on one lane of an Illumina HiSeq 2000 platform at a concentration of 8 pM.

### Bioinformatics analyses

The paired-end sequences from the four population samples were combined, stripped of terminal primers using a local script based on the NCBI Vector Screen tool [32] and assembled with ABySS [33] and Soapdenovo-Trans [34] using k parameters from 25 to 95 at intervals of 5, and Trinity [35]. The assemblies were joined using a iterative blast and CAP3 pipeline as previously described [36]. The results of these analyses were then piped into a hyperlinked Excel report, as described in the dCAS software tool [37]. The coding sequences (CDS) that encoded putative polypeptides of at least 40 amino acids (aa) were extracted according to i) matches to proteins in the NCBI nr database; ii) the largest ORF. Functional annotations of the transcripts were performed using the program Classifier (Ribeiro, unpublished), which combines the output of several tools: BLASTX to compare the nucleotide sequences to the NCBI nr protein database, Swissprot; rpsblast to search for conserved protein domains in Pfam, SMART, KOG, Conserved Domains Databases (CDD) and GO databases. Transcripts were also compared to mitochondrial and rRNA nucleotide sequences from NCBI.

Bowtie2 version 2.1.0 was used to index the complete assembled transcriptome derived from the pooled reads from the four populations. RNA-seq paired-end reads for each population were mapped independently using TopHat2 2.0.9 [38] against the combined transcriptome contigs with default parameters except for a maximum number of 3 mismatches. The resulting BAM format alignments were used as input for Cufflinks 2.1.1 [39]. Significance tests for differential transcript abundances were determined using Cuffdiff with a false discovery rate (FDR) of 0.05. The intersections among the four population samples (i.e. more abundant transcripts in one or more samples in comparisons with the other samples) of the antennal genes were visualized by a Venn diagram [40]. The Cuffdiff results were also visualized and explored using CummeRbund v. 2.0.0 [41] and additional statistical analyses were performed using R version 3.6.2 [42]

Gene ontology analysis was performed with Blast2GO [43]. Over- and under-representation of multiple-level GO categories [44] within the over-abundant transcripts identified by the Cuffdiff pair-wise analyses was evaluated using the Fisher’s Exact Test within Blast2GO (Enrichment analysis) with a false discovery rate (FDR) of 0.05.

To determine the phylogenetic relationships of odorant binding protein (OBP) and odorant receptor (OR) sequences encoding at least 100 or 150 amino acids, respectively, with their *Ae. aegypti* counterparts, the amino acid sequences (excluding signal peptide sequences, where present) were aligned using MAFFT v7 [45] with the E-INS-i strategy, BLOSUM62 matrix, 1000 maxiterate and offset 0. Phylogenetic relationships were estimated using Maximum Likelihood with 1000 bootstrap replications with MEGA 6.0.6 [46] and mid-point rooted trees were plotted using FigTree v1.4 [47].

Transmembrane domains in identified odorant receptor protein sequences were predicted using MEMSAT3 [48] and snake plots of the predicted structures were generated using TOPO2 [49].

SNP analyses were performed in accordance with the GATK best practices for variant calling on RNAseq [50]. The TopHat2 BAM files were sorted with respect to the *de-novo* reference transcriptome using the ReorderSam tool in Picard 1.108 [51], read groups were added, duplicates marked, and indexes were created using the Picard tools and reassignment of mapping qualities was performed using SplitNCigarReads tool in GenomeAnalysisTK (GATK 3.0–0) [52]. Variant calling was performed simultaneously for the four population libraries using the GATK HaplotypeCaller tool with a minimum phred-scaled threshold of 20. Variant filtering was performed using the GATK VariantFiltration tool to screen Fisher Strand values (FS > 30), quality by depth values (QD < 2.0) and clusters of at least 3 SNPs within a window of 35 bases. The Fisher Strand values are an assessment of strand bias (the variation being seen on only the forward or only the reverse strand) where more bias is indicative of false positive calls. The predicted effects of the SNP variants (synonymous/nonsynonymous) on the putative amino acid product were determined manually using CLC Main Workbench 6 (Qiagen) and MEGA 6.0.6. The numbers of fixed or polymorphic non-synonymous/synonymous sites relative to *Aedes aegypti* were used to assess the selective pressures acting on genes using the McDonald-Kreitman test [53] using DnaSP 6.12.03 [54] considering sites with a minimum coverage of 10 reads in the sorted, indexed BAM files as determined using Integrative Genomics Viewer (IGV) 2.5.3 [55]. Additional statistical analyses were performed using R version 3.6.2 [42].

### Reverse transcriptase-PCR (RT-PCR) for the analysis of the tissue-specificity of OBP/OR transcripts

Total RNA was extracted using Trizol (Invitrogen) from different body compartments of 2–4 day old male and female *Ae. albopictus* that had been maintained together in the same cage since emergence. Pools of each of the following were used: antennae (~ 100 pairs), palps (~ 100 pairs), proboscises (25), heads without antennae, palps and proboscises (5), tarsi (~ 10 sets), legs without tarsi (~ 10 sets), thoraces without wings and legs (5), abdomens (5) and wings (50 pairs). After DNAse treatment (DNAfree, Ambion), RNA integrity was determined by formaldehyde agarose gel electrophoresis and quantified using a Nanodrop ND-1000 spectrophotometer (Nanodrop Technologies). For each body part 200 ng of the extracted total RNA was transcribed into cDNA using the iScript™ cDNA Synthesis Kit (Biorad). RT-PCRs with gene specific primers, designed using Primer3Plus version 2.3.6 [56](Additional file 1: Table S1) were performed using 5% of the synthesized cDNA and the following cycle conditions: 94 °C for 3 min, 30 cycles at 94 °C for 30 s, 58 °C for 30 s, 72 °C for 2 min, and a final extension at 72 °C for 10 min. The *Ae. albopictus RpL34* gene (GenBank accession no. AF144549) was amplified as a control for cDNA integrity. To control for genomic DNA contamination, RT-PCR was also performed on samples in which cDNA synthesis had been performed in the absence of reverse transcriptase. The amplification products were electrophoresed on 2% agarose gels.

### Validation of RNAseq with real-time quantitative PCR

Real-time quantitative PCR (qRT-PCR) was performed on six genes (*Aalb-no mechanoreceptor potential C* (*nompC*), a *carboxylesterase* (*Aalb-CCEae3a*), a *cytochrome P450* (*Aalb-cyp450*) and three odorant binding proteins, *AalbOBP17* (Aalb-6031), *AalbOBP62* (Aalb-88,196) and *AalbOBP75* (Aalb-4806) using cDNA derived from the antennae of 2 to 3 day-old male and female individuals from lines derived from Trento (F_4_), Athens (F_3_) and Ban Rai (F_2_). Two reference genes, *Aalb-G6PDH* (Aalb-91,038) and *Aalb-RpL34* (GenBank accession no. AF144549) were used for relative quantification normalization [57] (Additional file 2: Table S2). *RpL34* has been used as a reference gene in this species in several previous studies [58, 59]. Synthesis of cDNA was performed using 100 ng RNA in 20 μl reaction volumes using the iScript cDNA Synthesis kit (Bio-Rad). Real-time quantification was performed using the SsoFast EvaGreen Supermix kit (Bio-Rad) and MasterCycler realplex (Eppendorf). Cycling conditions involved an initial 95 °C for 30s, 40 cycles of 10 s at 95 °C, 30 s at 58 °C. A fluorescence reading was made at the end of each extension step. Three biological replicates were performed, and the specificity of the amplification products was assessed by melt-curve analysis. Statistical comparison of RNAseq and qRT-PCR (log2 ratio) datasets was performed using Pearson correlation analysis [42].

## Results

### Differential transcription in the antennae of mosquitoes from different geographic populations

**Fig. 1:**
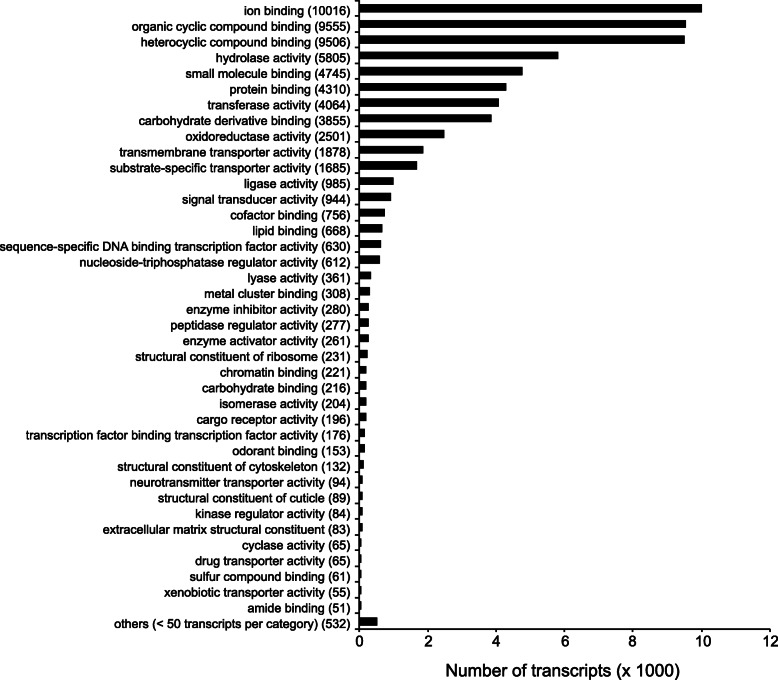
Distribution of the antennal assembled sequences in Gene Ontology Molecular Function categories level III

Over 420 million reads were generated from the antennae of male and female mosquitoes from the native Thai (Ban Rai, 140.6 M) and three adventive Mediterranean populations, Greece (Athens, 105.9 M) and Italy (Arco, 64.6 M and Trento, 109.0 M). The reads were assembled into 98,534 contigs with an N50 of 1296 bp. BLASTN of these contigs against both the Fellini and Foshan genomes [60, 61] resulted in hit rates of 95% (4464 and 4999 contigs, respectively, gave no hits with an expectation, *e* < 10^− 6^). When queried against the protein nr database and categorized into different gene ontology (GO) functional classes, the most frequent level III terms were ion binding, organic cyclic compound binding, heterocyclic compound binding, protein binding and small molecule binding (Fig. 1).

**Fig. 2:**
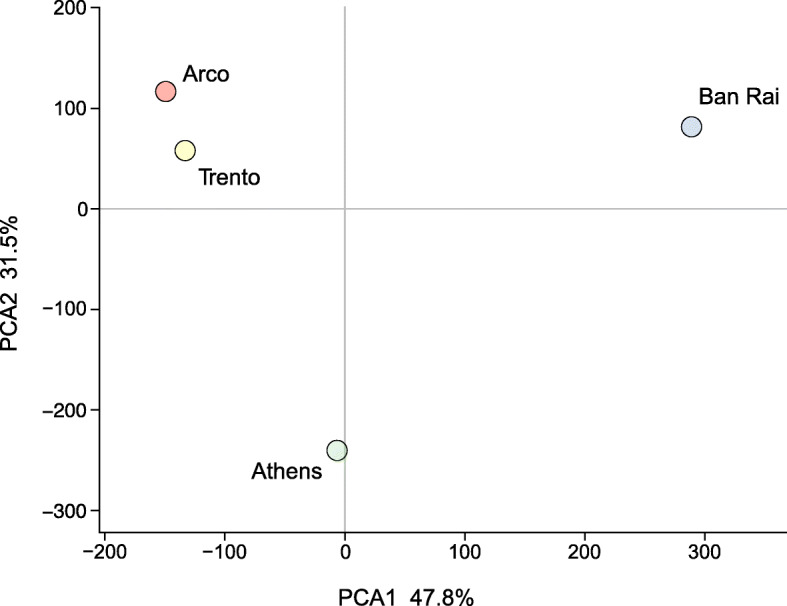
Principal Component Analysis based on transcript abundance illustrating the relationships between the four population samples

The four population samples, based on transcription levels (FPKM), were highly significantly different (paired Wilcoxon-Pratt signed rank test with continuity correction, *P* < 0.0001). This is evident also in a Principal Component Analysis (PCA, prcomp package, R) where component 1 (accounting for 47.8% of the variation), separates the native Thai population from the other three adventive populations, especially the two Italian populations. The second component (accounting for 31.5% of the variation) effectively separates the Athens population from the two Italian populations (Fig. 2). This differentiation of the populations was evident as 7418 of the transcripts displayed differential abundances in the four population samples with a False Detection Rate (FDR) of 0.05. These differentially abundant transcripts belong to a wide range of GO terms, (Additional file 3: Figure S1) including ion binding, organic cyclic binding, protein binding and odorant binding terms.

**Fig. 3:**
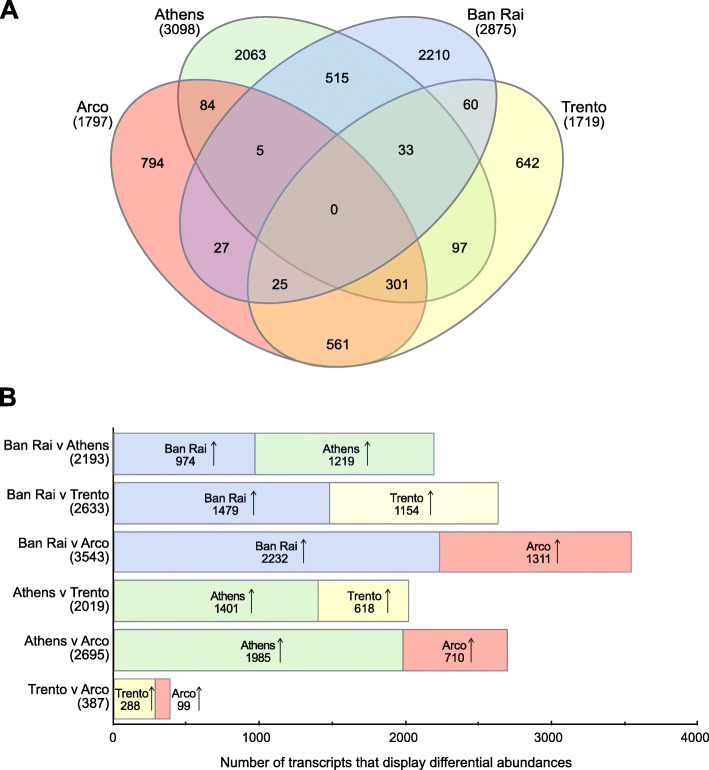
Differential abundances of transcripts in the different population samples. **a** Venn diagram illustrating the number of more abundant transcripts shared in the different population samples. **b** Numbers of transcripts that displayed differential abundances in the pairwise comparisons between the population samples. Arrows indicate that transcript abundance was greater in the respective population sample

Significantly more abundant transcripts in the different population samples and those shared between the different samples are shown in the Venn diagram (Fig. 3a). The Athens and the Ban Rai populations have the highest number of unique more abundant transcripts (2063 and 2210, respectively) and share a total of 553 more abundant transcripts of which 515 are shared only by them. Lower numbers of unique more abundant transcripts were present in the two Italian Arco and Trento populations (794 and 642, respectively) and they alone share 561 transcripts.

The number of transcripts with differential abundances varied greatly in the different population pairwise comparisons (Fig. 3b). The comparison between the native Thai (Ban Rai) sample and Arco resulted in the most differential abundances (3543 transcripts), with 37% more abundant in Arco and 63% in Ban Rai. Whereas, the two geographically related north Italian populations, Arco and Trento, showed the lowest number of differential abundances (387 transcripts), 26% of which were more abundant in Arco and 74% in Trento.

The nature of the antennal transcriptional differences between the population samples was explored by Enrichment analyses. When compared to the complete dataset there was significant over- and under-representation of GO categories among the differentially abundant transcripts present in many pairwise comparisons of the samples. The terms that were enriched in the different comparisons are summarised in Table 1. Full details of the terms are given in Additional file 4: Tables S3-S8.

**Table 1:** Enriched Gene Ontology categories in the different *Ae. albopictus* population sample comparisons

Comparison	Population	Summary of enriched GO categories
Arco v Trento	Arco	Regulation and maintenance of photoreception
	Trento	–
Athens v Trento	Athens	Nitrogen assimilation; oxidative processes
	Trento	Viral attachment and entry, viral genome replication
Athens v Ban Rai	Athens	Iron-ion binding
	Ban Rai	–
Ban Rai v Trento	Ban Rai	Nitrogen assimilation; oxidative processes
	Trento	Viral attachment and entry, viral genome replication
Arco v Athens	Arco	Viral attachment and entry, viral genome replication; odorant binding; photo transduction
	Athens	Nitrogen assimilation; oxidative processes; perception of sound
Arco v Ban Rai	Arco	Viral attachment and entry, viral genome replication; odorant binding; photo transduction; oxidative processes
	Ban Rai	Perception of sound, heat and smell; flight behaviour

As a confirmation of the validity of the RNA-seq transcript abundance estimates, a highly significant positive correlation (r = 0.9119, *P* < 0.0001) was found between the RNAseq and qRT-PCR datasets derived from the abundance of six transcripts in three population samples, Trento, Athens and Ban Rai (Additional file 5: Figure S2, Additional file 6: Table S9).

### Functional categories of antennal transcripts that are differentially abundant in the four populations

#### Sensory perception of smell

Functional categories related to odorant perception and sensory perception of smell were found to be overrepresented in the pairwise population antennal comparisons. Out of a total of 162 transcripts with significant similarity to odorant binding proteins (OBPs), 32 were identified as members of the Classic subfamily and 12 of the Plus-C subfamily [62] (Table 2, Additional file 7: Figure S3, Additional file 8: Table S10). No members of the Two-domain subfamily were identified. A repertoire of 242 transcripts shared significant sequence similarity with known odorant receptor (OR) proteins (e < 10^− 6^) from other species, and particularly from *Ae. aegypti* [63, 64]. Out of the considered 80 transcripts (Table 3), 49 encoded complete ORs ranging in size from 375 to 479 amino acids, whereas two appeared to be pseudogenes (Aalb-5361 and Aalb-81,229) as, despite being full length, they contained an internal stop codon or a frame-shift, respectively (Additional file 9: Figure S4, Additional file 8: Table S11).

**Table 2:** *Aedes albopictus* putative odorant binding protein (OBP) transcripts

			*Ae. aegypti* orthologue		Signal Peptide			
Transcript	aa	Name	Gene	Name	Pos, D	Subfamily		Complete^a^
Aalb-88,094	143	AalbOBP1	AAEL009449	AaegOBP1	18, 0.735	Classic	OS-E/OS-F	
Aalb-89,777	159	AalbOBP2	AAEL006176	AaegOBP2	24, 0.752	Classic	Pbprp1	
Aalb-96,031	143	AalbOBP3	AAEL013018	AaegOBP3	18, 0.747	Classic	OS-E/OS-F	
Aalb-88,146	134	AalbOBP4	AAEL000073	AaegOBP4	26, 0.747	Classic	OBP19a	
Aalb-4635	132	AalbOBP9	AAEL002596	AaegOBP9	20, 0.643	Classic	mclassic3	
Aalb-90,263	140	AalbOBP10	AAEL007603	AaegOBP10	25, 0.890	Classic	mclassic6	
Aalb-88,450	137	AalbOBP11	AAEL002587	AaegOBP11	18, 0.901	Classic	mclassic3	
Aalb-97,431	132	AalbOBP12	AAEL002617	AaegOBP12	18, 0.850	Classic	mclassic3	
Aalb-88,277	133	AalbOBP13	AAEL002591	AaegOBP13	18, 0.873	Classic	mclassic4	
Aalb-25,065	136	AalbOBP15	AAEL002598	AaegOBP15	23, 0.833	Classic	mclassic1	
Aalb-6031	138	AalbOBP17	AAEL004339	AaegOBP17	19, 0.813	Classic	mclassic7	
Aalb-98,240	141	AalbOBP18	AAEL004342	AaegOBP18	22, 0.927	Classic	mclassic9a	
Aalb-1010	125	AalbOBP19	AAEL004343	AaegOBP19	–	Classic	mclassic9a	N
Aalb-51,548	142	AalbOBP19-N1	AAEL004343	AaegOBP19	20, 0.902	Classic?	–	
Aalb-88,295	166	AalbOBP20/59	AAEL005778	AaegOBP20	24, 0.762	Classic	Pbprp4	
Aalb-2021	138	AalbOBP22	AAEL005772	AaegOBP22	16, 0.775	Classic	Obp99a	
Aalb-57,665	101	AalbOBP26/23	AAEL006109	AaegOBP23	–	Plus-C	mplus11	N, C
Aalb-10,170	200	AalbOBP25/24	AAEL006103	AaegOBP25	20, 0.796	Plus-C	mplus11	
Aalb-95,920	139	AalbOBP27	AAEL000071	AaegOBP27	19, 0.903	Classic	OBP19a	
Aalb-88,172	149	AalbOBP34	AAEL014082	AaegOBP34	24, 0.865	Classic	LUSH	
Aalb-86,836	131	AalbOBP35	AAEL002606	AaegOBP35	18, 0.904	Classic	mclassic3	
Aalb-91,258	152	AalbOBP36	AAEL008011	AaegOBP36	20, 0.795	Classic	OS-E/OS-F	
Aalb-94,240	149	AalbOBP37	AAEL008009	AaegOBP37	20, 0.940	Classic	OS-E/OS-F	
Aalb-90,197	140	AalbOBP38	AAEL008013	AaegOBP38	16, 0.883	Classic	OS-E/OS-F	
Aalb-91,445	146	AalbOBP39	AAEL006454	AaegOBP39	21, 0.719	Classic	LUSH	
Aalb-88,422	157	AalbOBP63/42	AAEL010666	AaegOBP42	No	Plus-C	mplus1	
Aalb-88,397	191	AalbOBP47	AAEL011499	AaegOBP47	20, 0.909	Plus-C	mplus1	
Aalb-87,394	151	AalbOBP55	AAEL012377	AaegOBP55	21, 0.690	Classic	OBP19a	
Aalb-88,160	140	AalbOBP56	AAEL000051	AaegOBP56	19, 0.601	Classic	OBP19a	
Aalb-92,750	155	AalbOBP59-N1	AAEL015313	AaegOBP59	21, 0.812	Classic	Pbprp4	
Aalb-88,453	122	AalbOBP60	AAEL015499	AaegOBP60	18, 0.877	Classic	OS-E/OS-F	C
Aalb-88,196	193	AalbOBP62	AAEL015566	AaegOBP62	21, 0.764	Plus-C	mplus1	
Aalb-89,129	181	AalbOBP69	AAEL000124	AaegOBP69	24, 0.816	Plus-C	mplus7	
Aalb-74,008	156	AalbOBP72	AAEL004729	AaegOBP72	–	Plus-C	mplus8	N
Aalb-57,651	166	AalbOBP73	AAEL004730	AaegOBP73	21, 0.780	Plus-C	mplus8	N
Aalb-45,154	169	AalbOBP73-N1	AAEL004730	AaegOBP73	23, 0.813	Plus-C	mplus8	
Aalb-4806	193	AalbOBP75	AAEL011483	AaegOBP75	19, 0.748	Plus-C	mplus5	
Aalb-96,930	133	AalbOBP76	AAEL007604	AaegOBP76	17, 0.765	Classic	mclassic6	
Aalb-46,119	120	AalbOBP77	AAEL002626	AaegOBP77	–	Classic	mclassic2	N, C
Aalb-13,742	116	AalbOBP78-N1	AAEL001836	AaegOBP78	–	Classic	*Bombyx mori*	N
Aalb-45,987	151	AalbOBP81	AAEL011730	AaegOBP81	23, 0.747	Classic	mclassic8	
Aalb-92,539	306	AalbOBP83	AAEL011416	AaegOBP83	29, 0.925	Classic	–	
Aalb-23,731	197	AalbOBP-N1	–	–	27, 0.679	Plus-C	?	
Aalb-17,964	103	AalbOBP-N2	AAEL004721	hypothetical protein	–	Plus-C	mplus4	N

^a^ N, C: incomplete at N- and/or C-terminus

**Table 3:** *Aedes albopictus* putative odorant receptor (OR) transcripts

			*Ae. aegypti* orthologue		Conserved Domains		
Transcript	aa		Gene	Name^a^	Domain	e-value	Incomplete^b^
Aalb-16,280	376	AalbOR2	AAEL005999	OR2	7tm_6	7.27E-47	
Aalb-5943	406	AalbOR4/5	AAEL015147	OR4	7tm_6	9.72E-43	
Aalb-82,460	401	AalbOR6	AAEL017548	OR6	7tm_6	8.39E-37	
Aalb-88,204	479	AalbOR7/ORCO	AAEL005776	OR7/ORCO	7tm_6	5.66E-41	
Aalb-8503	375	AalbOR10	AAEL006003	OR10	7tm_6	3.27E-43	
Aalb-95,352	420	AalbOR11	AAEL011583	OR11	7tm_6	8.93E-31	
Aalb-83,056	389	AalbOR13	AAEL008368	OR13	7tm_6	9.68E-28	
Aalb-9257	390	AalbOR15	AAEL008448	OR15	7tm_6	4.23E-18	
Aalb-92,214	400	AalbOR18/19	AAEL017017	OR19	7tm_6	2.49E-13	
Aalb-84,246	396	AalbOR20	AAEL004966	OR20	7tm_6	5.72E-12	
Aalb-47,088	396	AalbOR21	AAEL017398	OR21	7tm_6	1.05E-23	
Aalb-17,244	385	AalbOR22/N1^c^	AAEL002479	OR22	7tm_6	1.57E-31	N
Aalb-5704	402	AalbOR23	AAEL001510	OR23	7tm_6	3.88E-16	
Aalb-16,397	395	AalbOR24	AAEL017557	OR24	7tm_6	3.29E-17	
Aalb-19,286	180	AalbOR24-N1	AAEL017557	OR24	–	–	C
Aalb-84,055	381	AalbOR25	AAEL003629	OR25	7tm_6	2.53E-18	
Aalb-85,326	401	AalbOR26	AAEL010428	OR26	7tm_6	4.03E-20	
Aalb-88,595	384	AalbOR29	AAEL001310	OR29	7tm_6	1.08E-23	N
Aalb-17,821	384	AalbOR30	AAEL010409	OR30	7tm_6	1.95E-17	
Aalb-84,635	403	AalbOR31	AAEL013217	OR31	7tm_6	9.78E-44	
Aalb-81,229	396	AalbOR33	AAEL017362	OR33	7tm_6	2.66E-36	
Aalb-4286	406	AalbOR39	AAEL005489	OR39	7tm_6	4.01E-42	
Aalb-10,769	391	AalbOR42^c^	AAEL000616	OR41	7tm_6	3.03E-20	
Aalb-80,037	386	AalbOR42-N1	AAEL003045	OR42	7tm_6	6.57E-22	
Aalb-15,665	385	AalbOR44	AAEL006465	OR44	7tm_6	6.17E-23	
Aalb-5267	381	AalbOR45	AAEL000613	OR45	7tm_6	9.44E-22	N
Aalb-18,518	207	AalbOR45-N1	AAEL000613	OR45	7tm_6	2.04E-12	N,C
Aalb-97,870	384	AalbOR47-N2	AAEL017079	OR47	7tm_6	1.40E-27	
Aalb-56,511	170	AalbOR47-N3	AAEL017079	OR47	–	–	N,C
Aalb-7041	257	AalbOR50-N1	AAEL010426	OR50	7tm_6	4.01E-03	C
Aalb-5118	421	AalbOR52	AAEL013507	OR52	7tm_6	5.40E-11	
Aalb-70,366	395	AalbOR55	AAEL010415	OR55	7tm_6	3.88E-17	
Aalb-1114	250	AalbOR59	AAEL001342	OR59	–	–	C
Aalb-69,237	375	AalbOR62	AAEL011796	OR62	7tm_6	4.34E-11	N
Aalb-4058	286	AalbOR63	AAEL000628	OR63	7tm_6	3.38E-24	N
Aalb-89,176	404	AalbOR63-N2	AAEL000628	OR63	7tm_6	1.01E-23	
Aalb-696	389	AalbOR66	AAEL017227	OR66	7tm_6	4.75E-18	
Aalb-47,052	386	AalbOR69	AAEL001221	OR69	7tm_6	2.53E-12	
Aalb-73,112	199	AalbOR70	AAEL001224	OR70	7tm_6	2.92E-04	N,C
Aalb-10,463	385	AalbOR70-N1	AAEL001224	OR70	7tm_6	7.75E-19	
Aalb-81,174	385	AalbOR70-N2	AAEL001224	OR70	7tm_6	2.71E-17	
Aalb-13,202	422	AalbOR71^c^	AAEL017564	OR18	7tm_6	3.75E-23	
Aalb-6552	412	AalbOR72	AAEL017129	OR72	7tm_6	5.63E-09	
Aalb-5361	412	AalbOR72-N1	AAEL017129	OR72	7tm_6	4.82E-06	S
Aalb-6097	225	AalbOR76	AAEL013418	OR76	7tm_6	6.51E-04	N,C
Aalb-9669	395	AalbOR79	AAEL013420	OR79	7tm_6	7.00E-07	
Aalb-3076	422	AalbOR80	AAEL017221	OR80	7tm_6	2.39E-05	
Aalb-97,462	423	AalbOR81	AAEL017305	OR81	7tm_6	1.43E-08	
Aalb-18,076	353	AalbOR83c	AAEL000628	OR63	7tm_6	3.74E-20	N
Aalb-83,353	410	AalbOR84^c^	AAEL004218	OR85	7tm_6	1.81E-3	N
Aalb-89,654	416	AalbOR85^c^	AAEL017043	OR84	7tm_6	5.89E-07	
Aalb-89,563	424	AalbOR87	AAEL017347	OR87	–	–	
Aalb-445	353	AalbOR87-N1	AAEL017347	OR87	7tm_6	8.59E-05	N
Aalb-89,562	288	AalbOR87-N2	AAEL017347	OR87	–	–	N
Aalb-92,688	408	AalbOR88	AAEL014197	OR88	–	–	
Aalb-84,524	123	AalbOR94	AAEL017201	OR94	7tm_6	2.01E-03	N
Aalb-53,071	172	AalbOR94-N1	AAEL017201	OR94	–	–	C
Aalb-50,344	283	AalbOR99	AAEL017236	OR99	–	–	N
Aalb-96,847	415	AalbOR100	AAEL011409	OR100	–	–	
Aalb-26,945	210	AalbOR101	AAEL017050	OR101	–	–	N
Aalb-9171	363	AalbOR102	AAEL017143	OR102	7tm_6	9.37E-03	N
Aalb-96,630	405	AalbOR104-N1^c^	AAEL016966	OR104	7tm_6	2.24E-05	
Aalb-17,596	150	AalbOR109	AAEL017028	OR109	–	–	N
Aalb-47,815	244	AalbOR110	AAEL016983	OR110	–	–	N,C
Aalb-97,176	421	AalbOR111^c^	AAEL017178	OR105	7tm_6	2.97E-03	
Aalb-97,175	420	AalbOR111-N1^c^	AAEL017178	OR105	–	–	
Aalb-82,645	418	AalbOR113	AAEL017123	OR113	–	–	
Aalb-84,373	157	AalbOR113-N2	AAEL017123	OR113	–	–	N,C
Aalb-5235	230	AalbOR115	AAEL017361	OR115	–	–	N
Aalb-45,911	169	AalbOR117-N1	AAEL017377	OR117	–	–	N
Aalb-15,754	415	AalbOR117-N2	AAEL017377	OR117	7tm_6	6.14E-03	N
Aalb-7651	160	AalbOR117-N3	AAEL017377	OR117	–	–	N,C
Aalb-56,571	417	AalbOR117-N4	AAEL017377	OR117	7tm_6	4.45E-04	
Aalb-53,133	421	AalbOR121	AAEL017104	OR121	7tm_6	4.93E-04	
Aalb-16,771	406	AalbOR122	AAEL013563	OR122	–	–	
Aalb-71,045	376	AalbOR123	AAEL017537	OR123	7tm_6	2.41E-08	
Aalb-84,576	311	AalbOR125	AAEL013893	OR125	7tm_6	1.92E-05	N
Aalb-86,662	416	AalbOR-N5	AAEL017014	–	7tm_6	9.49E-05	
Aalb-401	413	AalbOR-N6	AAEL017014	–	–	–	N
Aalb-3853	414	AalbOR-N7	AAEL010669	–	7tm_6	1.89E-39	

^a^ according to Bohbot and colleagues [64]

^b^ N, C: incomplete at N- and/or C-terminus; S: contains internal stop

^c^name modified to comply with Lombardo and colleagues [65]

Seven contigs, encoding polypeptides between 53 to 273 amino acids in length, shared significant sequence similarity with known gustatory receptor (GR) proteins (Additional file 10: Table S12).

**Fig. 4:**
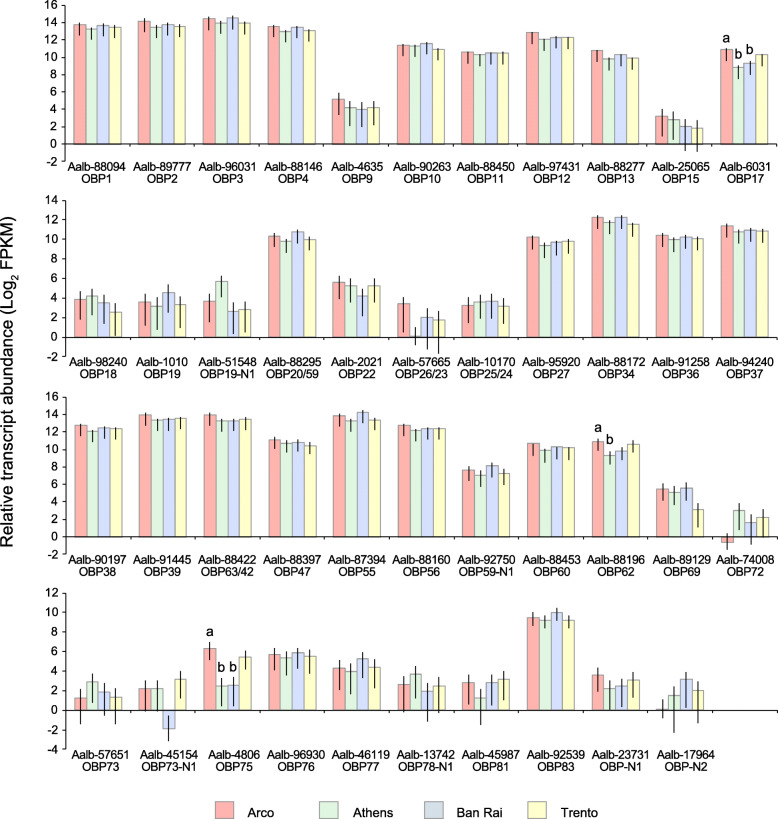
Odorant binding protein transcript abundances (Log_2_ FPKM) in the four wild population samples. 95% confidence intervals are displayed on each bar. Bars labelled with different letters indicate significant difference

**Fig. 5:**
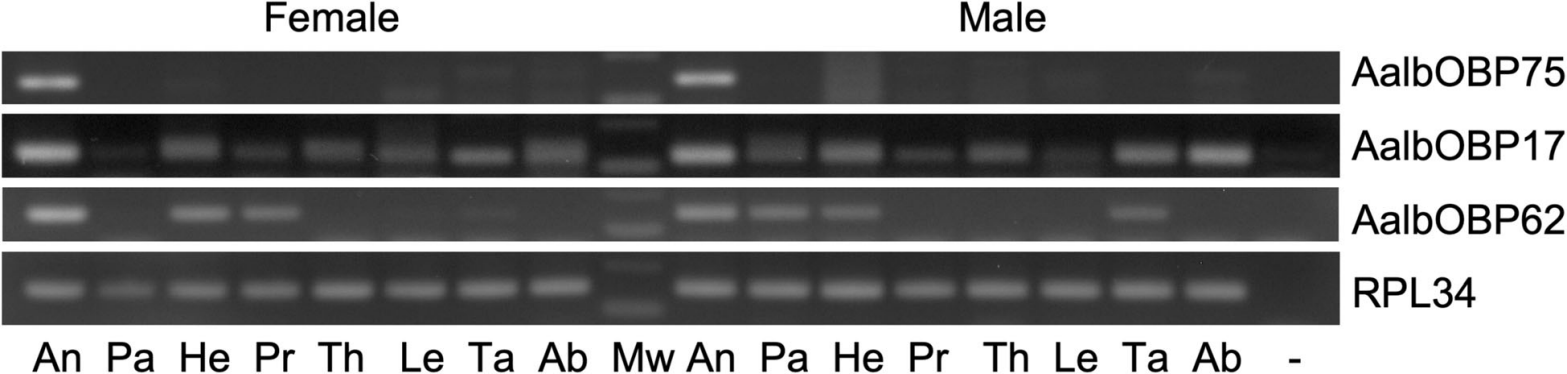
Transcriptional profiles of the three differentially transcribed *Ae. albopictus* OBP genes in different body parts of 2–3 day old males and females as determined by RT-PCR. An, antennae; Pa, palps; He, head minus antennae, palps and proboscis; Pr, proboscis; Th, thorax minus legs and tarsi; Le, legs minus tarsi; Ta, tarsi; Ab, abdomen; Mw, 100 bp DNA ladder (Invitrogen). The *RpL34* reference gene was amplified as a control for cDNA integrity

Within the OBP functional category three OBP transcripts were found to be differentially transcribed across the four population samples; the Classic subfamily Aalb-6031/AalbOBP17 member and two Plus-C subfamily members, Aalb-4806/AalbOBP75 and Aalb-88,196/AalbOBP62. The Classic AalbOBP17 was enriched 4.2-fold in Arco with respect to Athens (*P* = 0.0047) and 3-fold compared to Ban Rai (*P* = 0.0336). While the Plus-C AalbOBP75 was enriched 14-fold in Arco compared to both Athens and Ban Rai (*P* = 0.0389 and *P* = 0.0373, respectively). The other Plus-C OBP, AalbOBP62 was enriched 2.9-fold in Arco compared to Athens (*P* = 0.0189). The relative abundances of the OBP transcripts varied greatly and ranged from a mean FPKM value of 4.1 for Aalb-57,651/AalbOBP73 to a mean FPKM value of 19,539 for Aalb-96,031/AalbOBP3 (Fig. 4). Also, in terms of tissue specificity the three differentially abundant OBPs are heterogeneous. The Plus-C AalbOBP75 appeared to be specific for the antennae of males and females. The other Plus-C AalbOBP62 displays a wider tissue distribution also in relation to sexes; it is transcribed in the antennae, head and tarsi of both sexes but also in the female maxillary palps and proboscis. The Classic AalbOBP17 appeared to be transcribed in all body compartments, in both sexes, although the strongest signals were from the antennae (Fig. 5).

**Fig. 6:**
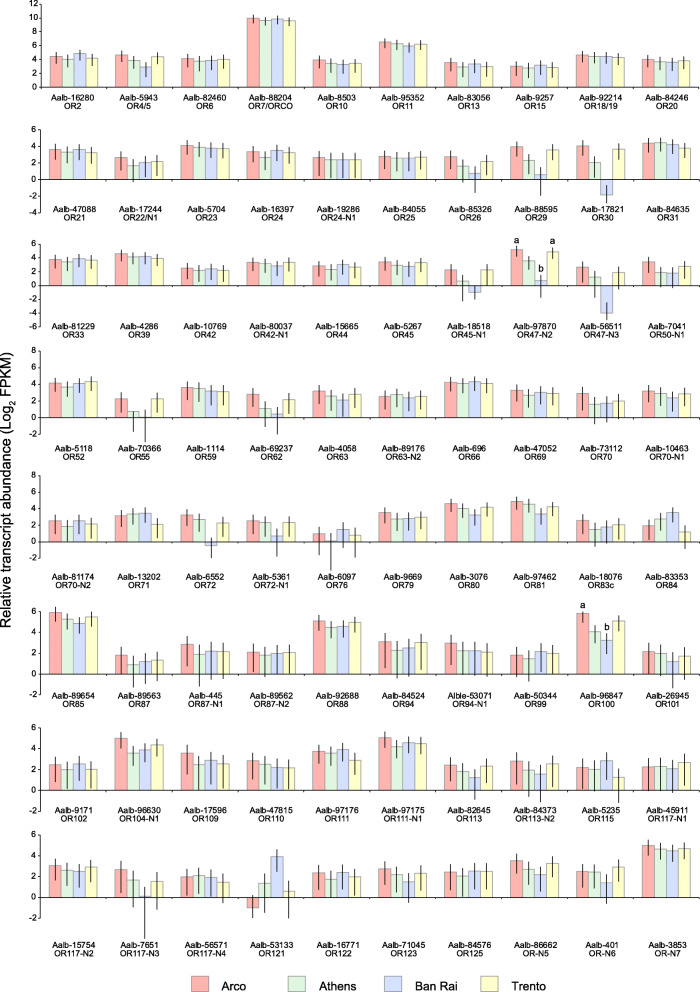
Odorant receptor transcript abundances (Log_2_ FPKM) in the four wild population samples. 95% confidence intervals are displayed on each bar. Different letters indicate significant difference

Within the OR functional category, two differentially abundant OR transcripts were identified across the four populations; Aalb-96,847/AalbOR100 was enriched 6 fold in Arco with respect to Ban Rai (*P* = 0.0174) while Aalb-97,870/AalbOR47-N2 was enriched 23 fold in Arco and 18 fold in Trento compared to Ban Rai (*P* = 0.0247 and *P* = 0.0247, respectively) (Fig. 6). The abundances of the OR transcripts differed enormously. The highest abundance (mean 868 FPKM in the four populations) was for AalbOR7/ORCO (Aalb-88,204), expected given its obligate co-expression with the ligand-specific tuning ORs. The other considered OR transcript abundances ranged from a mean FPKM value of 1.9 (Aalb-6097/AalbOR76) to 76.5 (Aalb-95,352/AalbOR11).

#### Viral infection terms

The over-representation of viral infection terms in the antennae of the north Italian populations, Arco and Trento compared to Athens and Ban Rai involved nine up-regulated sequences: Aalb-11,736, Aalb-24,050, Aalb-24,228, Aalb-50,498, Aalb-52,817, Aalb-67,423, Aalb-71,556, Aalb-77,037 and Aalb-82,640 which display very high identities (99–100%) with parts of the polyprotein gene of *Aedes* flavivirus (AeFV) (GenBank acc. KC181923 [66] GenBank acc. AB488408 [67]). These AeFV transcripts were abundant in the two Italian populations (average FPKM = 6.7 and 9.7 in Arco and Trento, respectively) and practically absent in the Athens (FPKM = 0.005) and Ban Rai (FPKM = 0.103) populations.

#### Iron-ion binding terms

Up-regulation of 14 cytochrome P450 transcripts, glutamate synthase, a carboxy/choline esterase, ABC transporter, aconitase and transferrin was found in Athens with respect to Trento and Thailand. Aalb-89,068, the homologue of AAEL005112, a Carboxy/cholinesterase alpha esterase, CCEae3a, showed up to 44-fold up-regulation in Athens (119.4 FPKM) compared to the other population samples (5.4 FPKM in Arco, 2.7 in Ban Rai and 4.1 in Trento).

#### Sound and visual perception terms

Transcripts associated with the perception of sound were enriched in Athens and Ban Rai in the comparisons with Arco. These include several dynein intermediate heavy chain homologues and a *nompC* (no mechanoreceptor potential C) homologue. The *nompC* homologue (Aalb-91,878) was significantly up-regulated (4.2 fold) in Ban Rai compared to Arco (17.6 versus 4.2 FPKM, respectively). The enriched regulation and maintenance of photoreception GO category in Arco in the comparisons with Trento and Athens involved three transcripts (Aalb-90,651, Aalb-94,114, Aalb-93,715) of two arrestins (homologues of *arrestin-1* and *arrestin-2* in *An. gambiae*).

### Single nucleotide polymorphism across the antennal transcriptomes

**Fig. 7:**
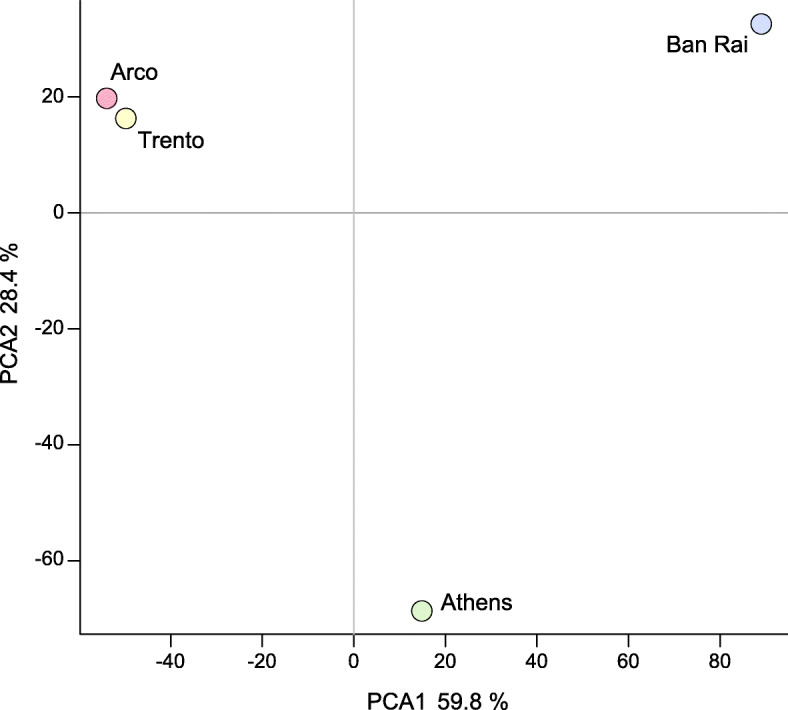
Principal Component Analysis based on biallelic SNP frequencies illustrating the relationships between the four population samples

A total of 958,216 GATK quality filtered SNP loci were identified in the assembled sequences, however almost 54% of these loci were clustered (three or more SNPs within a window of 35 bases) and when these were discarded the number of SNP loci fell to 441,230. The mean SNP densities (sites with at least one alternative allele/kb) were significantly different in the population samples (Ban Rai 10.35; Athens 9.08; Trento 9.17 and Arco 7.30, *P* = 0.0, bootstrap heteroscedastic one-way ANOVA for trimmed means (tiwaybt) followed by robust post hoc tests as implemented in the R package WRS2 [68]). The post hoc tests showed that the mean densities of the polymorphic sites were highly significantly different (*P* < 0.0001) in all but the Trento/Athens pairwise comparison (*P* = 0.2304). The polymorphic site densities were not correlated with read depth (FPKM) in the population samples (Pearson’s product-moment correlation: Arco, *r* = 0.0053, *P* = 0.237; Athens, *r* = − 0.0019, *P* = 0.662; Ban Rai, *r* = 0.0068, *P* = 0.1192; Trento, *r* = 0.0056, *P* = 0.2039) indicating that read coverage did not bias SNP detection (Additional file 11: Figure S5). A PCA (prcomp package, R) (Fig. 7) based on the allelic frequencies of 254,336 biallelic SNP loci (with a filtered read depth of at least 20 in each population) was congruent with that derived from transcript levels (Fig. 2) with the first component (accounting for 59.8% of the variation), grouping the Athens and native Ban Rai populations and effectively separating them from the two Italian populations. The second component (accounting for 28.4% of the variation) separates the Athens population from the native Ban Rai population.

#### SNP variation within the olfactory protein transcripts

The 44 considered OBP transcripts contain 463 SNP loci (including clustered loci). Most were bi-allelic, with only six tri-allelic loci (1.3%) in six transcripts: AalbOBP22, AalbOBP34, AalbOBP69, AalbOBP59-N1, AalbOBP60 and AalbOBP76. Over 18% (85) of the SNP variants result in non-synonymous substitutions, whereas the remaining variants result in synonymous substitutions. However, as 25 of the non-synonymous substitutions are in the signal-peptide encoding sequences, just under 13% (60) of the SNPs would result in amino acid substitutions in the mature OBPs (Additional file 12: Table S13).

**Fig. 8:**
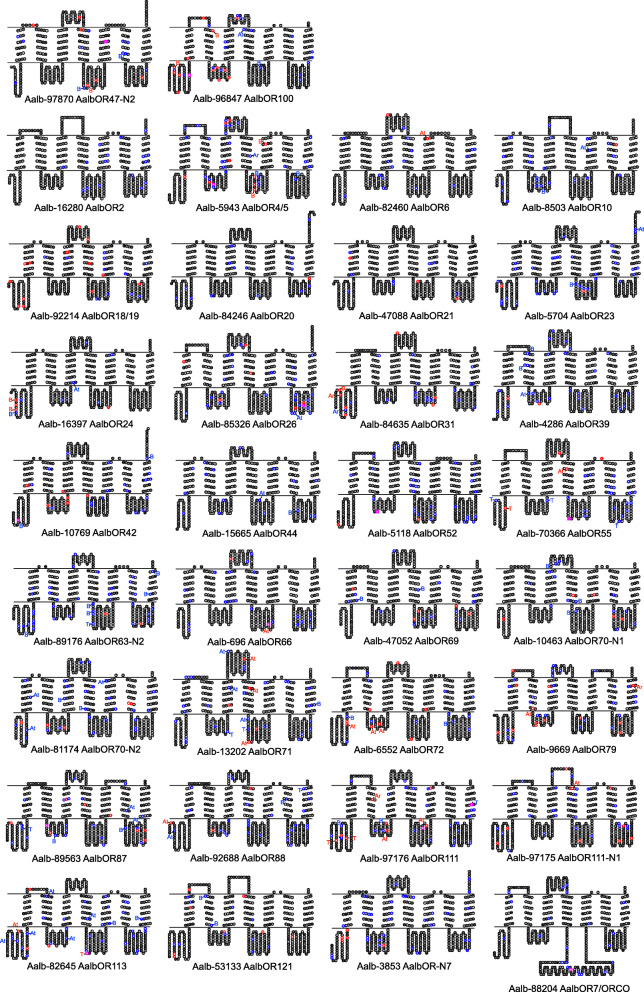
Snakeplots of *Ae. albopictus* ORs. Amino acids are coloured to indicate synonymous (blue), non-synonymous (red) and the presence of both non-synonymous and synonymous substitutions within the codon (magenta). Coloured circles indicate codons with one SNP locus, squares indicate codons with two or more SNP loci. The position of SNP variants that are private to one population sample (B = Ban Rai, At = Athens, Ar = Arco, T = Trento) are indicated

A total of 1900 SNP loci (including clustered loci) are present in the 78 OR transcripts, 16 of which are triallelic. About 25% (484) of the SNP variants result in non-synonymous substitutions (Additional file 13: Table S14). The differentially transcribed AalbOR100 contains 31 SNP loci, of which 13 result in non-synonymous substitutions. Four of these SNP variants are private, three in Ban Rai (two of which are non-synonymous) and one in Athens. The other differentially expressed odorant receptor, AalbOR47-N2 contains 19 SNP loci, seven of which are non-synonymous. Three of these SNP variants, including a non-synonymous substitution, were private, all in Ban Rai. In both these ORs, a non-synonymous substitution was present in the extracellular loop 2 (ECL2) region that has been shown to determine odorant specificity [69, 70] (Fig. 8). Of the other 32 OR proteins, for which it was possible to derive the seven transmembrane domain structure, ten contained from one to two non-synonymous substitutions in the ECL2 region. The AalbOR7/ORCO transcript has 23 SNP loci of which only one variant is non-synonymous resulting in the substitution of a Serine with another polar residue, Threonine, in the ORCO-specific insertion on the second intracellular loop (ICL2) of the protein complex [71, 72]. This substitution is private to the Ban Rai population with a frequency of 16%. The affected amino acid is 51 residues downstream from a calmodulin binding site (CaM) also in ICL2. The ORCO CaM binding site is involved in the phenomenon of sensitization, whereby repetitive subthreshold odour stimulation of olfactory sensory neurons sensitizes the OR/ORCO complex to give a super threshold response, enabling the insect to detect very faint odour traces [73, 74].

In contrast to these highly variable genes, the two arrestin genes (*arrestin-1* and *arrestin-2*) contained respectively 33 and 22 SNP loci. Only one of the SNP loci, in *arrestin-1*, was non-synonymous resulting in the substitution of a non-polar Phenylalanine with a polar Tyrosine. This polymorphism is within the arrestin N-terminal domain.

#### Tests of selection for the differentially transcribed OBP and OR transcripts

Evaluation of the ratios of fixed or polymorphic, non-synonymous/synonymous sites in the differentially transcribed OBP and OR genes, AalbOBP17, AalbOBP75, AalbOBP62, AalbOR47-N2 and AalbOR100, compared with their *Ae. aegypti* orthologues did not reveal any significant indication of departure from neutral molecular evolution (Fisher’s Exact test *P* > 0.05, McDonald-Kreitman test [53]).

## Discussion

Here we provide an overview of the vast reservoir of transcribed genes coding for sensory related functions in the antennae of wild mature adult mosquitoes collected in different ecogeographic areas. The information present in antennal transcriptomes is important both from an evolutive and an applicative point of view. Indeed, it is the basis to analyse and interpret different biological traits related to the behaviour and reproduction of this highly invasive mosquito. Another biologically important result is the high transcriptional variability of the sensory related genes among the wild populations of this mosquito. This variability may reflect the genetic plasticity that characterizes this mosquito and that has supported its high invasive potential. We have identified functional gene categories that display population-specific differential transcription levels. Given the public health impact of this mosquito, the information that we provide may prove important in the implementation of control methods such as the Sterile Insect Technique (SIT).

### Functional gene categories displaying population-specific differential transcription levels

#### Sound, vision and odorant perception terms

Enrichment of transcripts of genes associated with the perception of sound was evident in the Greek Athens and Thai Ban Rai samples in the comparisons with the Italian Arco sample. These transcripts included several dynein intermediate and heavy chain homologues and a *nompC* (no mechanoreceptor potential C) homologue which in *Drosophila* are expressed in chordotonal neurons. These are required for mechanical amplification of vibrations and mechanosensory function within the Johnston’s organ, a chordotonal organ near the base of the antenna [75]. In the mosquito, the Johnston’s organs are especially well developed in the male [76], where they are acutely sensitive to the wing-beat frequency of the female mosquito – a cue that is essential for mating success [77]. These organs may have additional functions such as gravitactic behaviour and the detection of air currents during flight [78].

The enriched regulation and maintenance of photoreception GO category in Arco compared to Trento and Athens involved homologues of *arrestin-1* and *arrestin-2* of *An. gambiae*. Arrestins are involved in the desensitization of both visual and olfactory transduction pathways where they regulate G protein-coupled receptors [79, 80]. Given the antennal origin of the transcriptome, these arrestins may be regulating olfactory signalling by coupling to ORs [64]. Thus, this GO-term may relate to olfaction rather than to vision.

Transcripts related to the perception of smell were enriched in Ban Rai compared to Arco. These included homologues of the sensory neuron membrane protein 1 (SNMP1), a member of a family of SNMPs originally identified in Lepidoptera where they are antennal-specific and are associated with pheromone specific sensilla [81]. In *D. melanogaster* SNMP1 is essential for the detection of the male-specific pheromone cis-vaccenyl acetate [82].

The apparent absence of many *Ae. aegypti* OBP, OR and GR homologues is not unexpected, as many will be associated with chemosensory tissues other than the antennae, such as the maxillary palps, proboscis and other parts of the body [83]. Indeed, the OBP and OR repertoires here identified differ slightly compared to those recently described in *Ae. albopictus* by Lombardo and colleagues [65], that analysed both antennae and maxillary palps (Additional file 8: Table S10). Other genes may be limited to larval stages [63], or different physiological states (e.g. after blood-feeding).

Among the identified OBP transcripts, three were significantly up-regulated in the Arco population sample in the comparisons with both the Athens and Ban Rai samples (AalbOBP17, AalbOBP75) or with Athens (AalbOBP62). The Classic OBP AalbOBP17 appears to be transcribed in numerous body compartments in *Ae. albopictus* whereas its orthologue AaegOBP17/AAEL004339 is mainly transcribed in the antennae of females and, to a lesser extent, in males [84]. The Plus-C OBP, AalbOBP75, appears to be transcribed only in the antennae of both sexes, whereas its *Ae. aegypti* orthologue (AaegOBP75/AAEL011483) is mainly transcribed in the female antennae (where blood-feeding appears to up-regulate its transcription), in maxillary palps and to a lesser extent in the female rostrum and male antennae [84]. Furthermore, AaegOBP75 is enriched in the salivary glands of virally naïve females [85]. The enriched AalbOBP62, another Plus-C OBP, has a transcriptional profile that is congruent with that shown by Deng and colleagues [86] and with that of its *Ae. aegypti* orthologue (AaegOBP62/AAEL015566) [84]. Interestingly, AaegOBP62 transcription increased in *Ae. aegypti* females 24 h after infection with yellow fever virus [87].

At least 83 ORs are transcribed in the antennae of *Ae. aegypti* [63], a number similar to that identified in the current study (at least 78). Due to its highly-fragmented state, the published draft Fellini genome sequence [60] was only marginally useful for assembly of the many OBP- and OR*-*like fragments. In contrast, the Chinese strain genomic sequence [61], though less fragmentary, appears to be rather redundant. Transcripts of two ORs, AalbOR100 and AalbOR47-N2, were found to be up-regulated in one or both Italian populations compared to the Ban Rai population. Both *Ae. aegypti* orthologues of these ORs are enriched in female compared to male antennae [84] and are upregulated in domestic forms that prefer to bite humans compared to forest forms that prefer guinea pigs [88], suggesting that they may be involved in the perception of odours related to host detection.

The identification of the highly conserved gustatory receptor GR1 orthologue (98% identity with AaegGR1) in the antennae is noteworthy, as in *Ae. aegypti*, this gene is expressed exclusively in the maxillary palps [89], where it acts as a CO_2_ receptor. Carbon dioxide is an important cue that emanates from potential blood-meal hosts and guides females over relatively long distances and, together with heat and other olfactory cues, stimulates blood feeding.

#### Iron-ion binding terms

The iron-ion-binding GO category was enriched in the Athens sample compared to Trento and Ban Rai, with 14 cytochrome P450 transcripts up-regulated, together with glutamate synthase, a carboxy/choline esterase alpha esterase, ABC transporter, aconitase and transferrin. Aconitase or iron regulatory protein 1 (IRP1) controls iron uptake by cells via the transferrin receptor and iron storage within cells as ferritin [90]. Transferrins have high affinities for iron and are involved in metabolism, immunity and development. Transferrins are up-regulated following bacterial or parasite infection and sequester iron from pathogens [85, 91]. Several studies have shown that transferrins are down-regulated in individuals infected with CHIKV and DENV-2 infections [87, 92], and it has been suggested that this may favour viral replication and survival, perhaps due to subversion of the insect’s pathways by the arboviruses [93].

Numerous cytochrome P450 transcripts were more abundant in the Athens sample. Cytochrome P450s are one of the largest and oldest gene superfamilies in insects and are involved in the metabolism of many endogenous and xenobiotic compounds including the detoxification of insecticides [94]. Indeed, at least four of the more abundant cytochrome P450s (CYP6Z8, CYP6Z9, CYP9J26 & CYP9J9) have been implicated in Deltamethrin and or Permethrin resistance in *Ae. aegypti* [95]. The extremely high abundance of Carboxy/cholinesterase alpha esterase, (AealbCCEae3a) in the Athens population (59-fold) compared to the other population samples is surprising. But, given that the *Ae. albopictus* CCEae3a gene has been implicated in temephos resistance, an organophosphate (OP) insecticide that has been used extensively as a larvicide in the Athens area for many years [95, 96], it is probable that this upregulation, due to gene copy number amplification, is more likely related to insecticide resistance than to iron-ion-binding. Recently a study of population samples from 16 countries found evidence of amplification only in Athens and Florida [58]. Two independent amplification types were identified, one involving co-amplification of the linked CCEae3a and CCEae6a genes, and individuals with this type in Athens and Florida shared the same haplotype. The second amplification type involves only the CCEae3a gene and was found only in Florida.

#### Nitrogen assimilation terms

Another up-regulated category related to nitrogen assimilation included glutamate synthase activity. Ammonia is metabolised, primarily in the fat body, by glutamate synthase with the production of glutamine and of proline that can be used as an energy source for flight [97, 98]. Why this term should be enriched in the Athens and Ban Rai samples compared to the two Italian samples is not clear. It should, however be mentioned that the adventive Athens mosquitoes share a common demographic history with those from Thailand [18].

#### Viral infection terms

The enrichment of viral infection terms in the two geographically close northern Italian populations related to the polyprotein gene of an *Aedes* flavivirus (AeFV). This virus was initially identified in Japanese populations of *Ae. albopictus* and *Ae. flavopictus* collected in 2003–2004 [66] and subsequently in *Ae. albopictus* from Missouri, USA in 2011 [67] and *Ae. albopictus* from Curitiba, Brazil in 2016 [99]. AeFV appears to be an insect-specific flavivirus (ISF) with no known vertebrate host, and it has been shown to be transmitted vertically to progeny [67]. AeFV has been shown to be widely distributed in *Ae. albopictus* in northern Italy including the Arco and Trento populations with an estimated viral infection prevalence of 16.84% in the Trentino province [100–102] and 3.12% in Veneto [102]. The northern Italian *Ae. albopictus* populations were established through a mixture of genomes originating from introductions from North America, the Pacific area and Japan/Southeast Asia [18, 19, 103, 104]. The Athens population, however, appears to be the result of a recent introduction directly from Thailand [18, 19]. The differences in the AeFV transcript abundances, high in the two Italian populations and practically absent in the Thai and Athens samples, may thus reflect the historical demographic relationships between the population samples. However, the possibility of de novo flavivirus infection of the Italian populations cannot be excluded. Whether these transcripts represent viral integrations in the mosquito genome or infections needs additional study. Should they derive from infections, it would suggest that the viral infection is maintained in the derived populations despite the bottleneck events that accompany new colonization events and the viral prevalence persists over numerous generations.

The presence of these non-pathogenic ISFs within a mosquito host may have important implications for disease dynamics [102]. It has been postulated that ISF infections may reduce the ability of the mosquito to harbour and transmit other related disease-causing viruses as a result of competition between the viruses for the host, due to ‘super-infection exclusion’ [105, 106]. For instance, the higher prevalence of AeFV in Trentino compared to populations from Veneto may be correlated with the apparent lower incidence of West Nile virus and Usutu virus in the Trentino with respect to Veneto [102].

### A vast repertoire of SNPs is in present in the antennal transcriptomes of wild populations

The identification of nearly one million SNP loci represents a useful addition to the arsenal of high-resolution molecular markers available in this species (reviewed in [16, 18]). Indeed, unlike *Anopheles gambiae* [107] and *Aedes aegypti* [108], few high-resolution molecular markers such as single-nucleotide polymorphisms (SNPs) were previously available for *Ae. albopictus* [16, 21–23]. The availability of a large number of SNP markers distributed throughout the genome is advantageous as it permits the identification of fine-scale differentiation between populations. Furthermore, given their transcriptomic origin, these SNPs may represent markers of selective pressure and adaptations that have occurred during the colonization of new habitats. The SNP densities in the four population samples ranged from 7.30 SNPs per kilobase in Arco to 10.35 in Ban Rai. The significantly higher SNP density and frequency of private SNP variants in Ban Rai (Additional file 13: Table S14) is a further indication of the native status of this population. SNP loci were abundant in the OBP and OR transcripts with numerous non-synonymous variants that may, depending on their position and the properties of the amino acids involved, change the affinity of the protein for its ligand.

## Conclusions

The high degree of inter-population transcriptional diversity highlights, at the functional level, the remarkable genetic flexibility of this mosquito species. We can hypothesize that the differential expression of genes, including those involved in sensory perception, in different populations may enable *Ae. albopictus* to exploit different environments and hosts. Furthermore, the discovery of this vast reservoir of transcriptional variability can help us interpret, at the molecular/functional levels, some of the biological traits that support its invasive potential.

From an evolutive point of view, in relation to the invasion process, the native Thai population appears to be the most differentiated with respect to the adventive Mediterranean populations, which are representative of some of the oldest invasion events outside Asia [18]. Among these Mediterranean populations, Athens is quite apart and is unique in sharing with Thailand a high number of enriched transcripts. This may be expected considering its direct demographic origin from Thailand [18, 19], while the origins of the northern Italian Arco and Trento populations is more complex [10, 18, 21]. Thus, we can speculate that ancestry, consequent to the demographic histories of populations, may affect the transcriptional profiles of genes related to sensory perception, which in turn may influence their behaviour.

A large number of SNP loci are also present in these transcripts across the considered populations. SNP variation within these genes may highlight the effect of the colonization processes and may reveal details of *Ae. albopictus* movement and gene flow patterns. This is evident in the analysis of SNP frequencies in the four considered populations where the relationships between the native Thai population, the adventive Athens and Italian populations was supported. Here, given the antennal origin of the libraries, we have highlighted SNP variation in a subset of genes involved in chemoreception, but clearly the resource we have developed can be applied to the study of a vast array of functional categories. These transcriptome-derived SNPs represent an important addition to the largely non-coding region-derived SNPs previously identified [10, 21–23].

## Supplementary information

**Additional file 1: Table S1.** Primers used for RT-PCR analyses.

**Additional file 2: Table S2.** Primers used for qRT-PCR analyses.

**Additional file 3: Figure S1.** Molecular function (level 3) Gene Ontology classification of the transcripts that were differentially abundant in the different population libraries.

**Additional file 4: Table S3.** Significantly over- and under-represented gene ontology categories among transcripts that showed changes in abundance in Ban Rai compared to Athens. **Table S4.** Significantly over- and under-represented gene ontology categories among transcripts that showed changes in abundance in Ban Rai compared to Trento. **Table S5.** Significantly over- and under-represented gene ontology categories among transcripts that showed changes in abundance in Ban Rai compared to Arco. **Table S6.** Significantly over- and under-represented gene ontology categories among transcripts that showed changes in abundance in Athens compared to Trento. **Table S7.** Significantly over- and under-represented gene ontology categories among transcripts that showed changes in abundance in Athens compared to Arco. **Table S8.** Significantly over- and under-represented gene ontology categories among transcripts that showed changes in abundance in Arco compared to Trento.

**Additional file 5: Figure S2.** Validation of RNA-seq derived changes in transcript abundance with quantitative real-time RT-PCR for six genes. The log_2_ transformed ratios of FPKM in comparisons between population samples are plotted against the corresponding log_2_ transformed relative transcript abundance values obtained with real-time qRT-PCR ratios for six genes (Additional file 6: Table S9). The Pearson correlation coefficient, *r* = 0.912, is highly significant (*P* = 3.6E-05).

**Additional file 6: Table S9.** Correlation of RNAseq transcript abundance ratios with real-time qRT-PCR ratios for six genes (log_2_ transformed).

**Additional file 7: Figure S3.** Phylogenetic relationships of OBP proteins from *Ae. albopictus* and *Ae. aegypti*. The unrooted maximum likelihood (log likelihood = − 27,485) tree was inferred using the W&G model (Whelan and Goldman 2001) with a discrete Gamma distribution. Bootstrap values greater than 50% (1000 replications) are shown. OBPs belonging to the Classic, Plus-C and Two domains subfamilies are highlighted.

**Additional file 8: Table S10.** Comparison of the odorant binding protein transcripts identified against the Lombardo (2017) transcripts. **Table S11.** Comparison of the odorant receptor transcripts identified against the Lombardo (2017) transcripts.

**Additional file 9: Figure S4.** Phylogenetic relationships of OR proteins from *Ae. albopictus* and *Ae. aegypti*. The unrooted maximum likelihood (log likelihood = − 84,078) tree was inferred using the JTT model (Jones et al. 1992) with a discrete Gamma distribution and amino acid frequencies (JTT + G + F). Bootstrap values greater than 50% (1000 replications) are shown.

**Additional file 10: Table S12.***Aedes albopictus* putative odorant receptor (GR) transcripts (BLASTP against NR database).

**Additional file 11: Figure S5.** Relationship between the number of polymorphic sites per kilobase and read depth (FPKM) for the four population samples. For clarity, the x-axes are truncated at FPKM = 2000, which precludes the vision of less than 0.09% of the data points. Trendlines are plotted on each of the graphs.

**Additional file 12: Table S13.** Frequencies of synonymous and non-synonymous SNP variants in the OBP transcripts.

**Additional file 13: Table S14.** Frequencies of synonymous and non-synonymous SNP variants in the OR transcripts.

## Data Availability

The sequence data generated during this study is deposited on the NCBI Sequence Read Archive, under BioProject ID PRJNA602498 (Accessions SAMN13896421-SAMN13896424). The transcript assembly, abundance and SNP data have been submitted to the Open Science Framework data repository, (10.17605/OSF.IO/SJBVT).

## Abbreviations

aa: Amino acid
ABC: ATP-binding cassette
ABySS: Assembly by short sequences
AeFV: *Aedes* flavivirus
ANOVA: Analysis of Variance
BAM: Binary Alignment Map
BLAST: Basic Local Alignment Search Tool
BLOSUM: BLOcks SUbstitution Matrix
bp: Base pair
CaM: Calmodulin binding site
CAP3: Contig assembly program Version 3
CDD: Conserved Domains Databases
cDNA: complementary DNA
CDS: Coding sequence
CHIKV: Chikungunya virus
CO_2_: Carbon dioxide
dCAS: Desktop cDNA Annotation System
DENV-2: Dengue virus serotype 2
DNA: Deoxyribonucleic acid
DNAse: Deoxyribonuclease
DnaSP: DNA sequence polymorphism analysis
FDR: False discovery rate
FPKM: Fragments Per Kilobase of transcript per Million mapped reads
FS: Fisher Strand
G0: Generation zero
GATK: Genome Analysis ToolKit
GO: Gene ontology
GR: Gustatory receptor
ICL2: Second intracellular loop
IGV: Integrative Genomics Viewer
IRP1: Iron regulatory protein 1
ISF: Insect-specific flavivirus
KOG: EuKaryotic Orthologous Groups
M: Million
MAFFT: Multiple Alignment using Fast Fourier Transform
MEGA: Molecular Evolutionary Genetics Analysis
MEMSAT3: MEMbrane protein Structure And Topology Version 3
mRNA: Messenger ribonucleic acid
NCBI: National Center for Biotechnology Information
OBP: Odorant binding protein
OP: Organophosphate
OR: Odorant receptor
ORCO: Odorant receptor coreceptor
ORF: Open reading frame
PCA: Principal component analysis
PCR: Polymerase chain reaction
Pfam: Protein families database
qRT-PCR: Quantitative reverse transcription polymerase chain reaction
RNA: Ribonucleic acid
SIT: Sterile Insect Technique
SMART: Simple Modular Architecture Research Tool
SNMP: Sensory neuron membrane protein
SNP: Single nucleotide polymorphism
TOPO2: Topoisomerase based cloning vector
