# Supplementary material for: Transcriptional variation of sensory-related genes in natural populations of Aedes albopictus
Source: BMC Genomics. 2020 Aug 7;21:547. doi: 10.1186/s12864-020-06956-6 (PMC7430840; doi:10.1186/s12864-020-06956-6)
Supplement: Supplementary file 1 — Additional file 1: Table S1. Primers used for RT-PCR analyses. [file 12864_2020_6956_MOESM1_ESM.docx]

Table S1 Primers used for RT-PCR analyses

| Transcript | forward primer | reverse primer |
| --- | --- | --- |
| Aalb-6031 (AalbOBP17) | tgctgcaggaagtgaaatcg | agcactcgtacagcttgaac |
| Aalb-4806 (AalbOBP75) | gacaagaacccgaaggaatg | gccagacaattgtgatgtgg |
| Aalb-88196 (AalbOBP62) | agcttgtgggattgttgcag | tcagttgacattcggcgatg |
| AF144549 (RPL34) | aaggagaagctcagcggaatc | tcaggaatgcacggatgatg |
