# Supplementary material for: Transcriptional variation of sensory-related genes in natural populations of Aedes albopictus
Source: BMC Genomics. 2020 Aug 7;21:547. doi: 10.1186/s12864-020-06956-6 (PMC7430840; doi:10.1186/s12864-020-06956-6)
Supplement: Supplementary file 2 — Additional file 2: Table S2. Primers used for qRT-PCR analyses. [file 12864_2020_6956_MOESM2_ESM.docx]

Table S2 Primers used for qRT-PCR

| Sequence ID | forward primer | reverse primer |
| --- | --- | --- |
| Aalb-91878 (nompc) | aaacagtgcgaaacgatggc | aactccatgttgcgtcgatc |
| Aalb-4806 (AalbOBP75) | tgcatgtttgagcagcaagg | ttcgcaactgtttcccactc |
| Aalb-6031 (AalbOBP17) | cgcaaggcatcaaagaactgg | tccggtggaaacttcaactcc |
| Aalb-88196 (AalbOBP62) | agagcttgtgggattgttgcag | tgtggcgttcagttgacattcg |
| Aalb-87486 (cyp450) | ttcgatccggatcacttcttcc | aggcacgcaacaaatgacac |
| Aalb-89068 (CCEae3a) | attcggaaaaccgcacgatc | aagttgttgggttgcgatgg |
| AF144549 (RPL13) | aacaccaagtccaacaagcg | tgagcttctccttgcactgg |
| Aalb-91038 (G6PDH) | agcctggcgaattttcactc | tgttctcgtcgcacttcttg |
