# Supplementary figures and images for: Transcriptional variation of sensory-related genes in natural populations of Aedes albopictus
Source: BMC Genomics. 2020 Aug 7;21:547. doi: 10.1186/s12864-020-06956-6 (PMC7430840; doi:10.1186/s12864-020-06956-6)

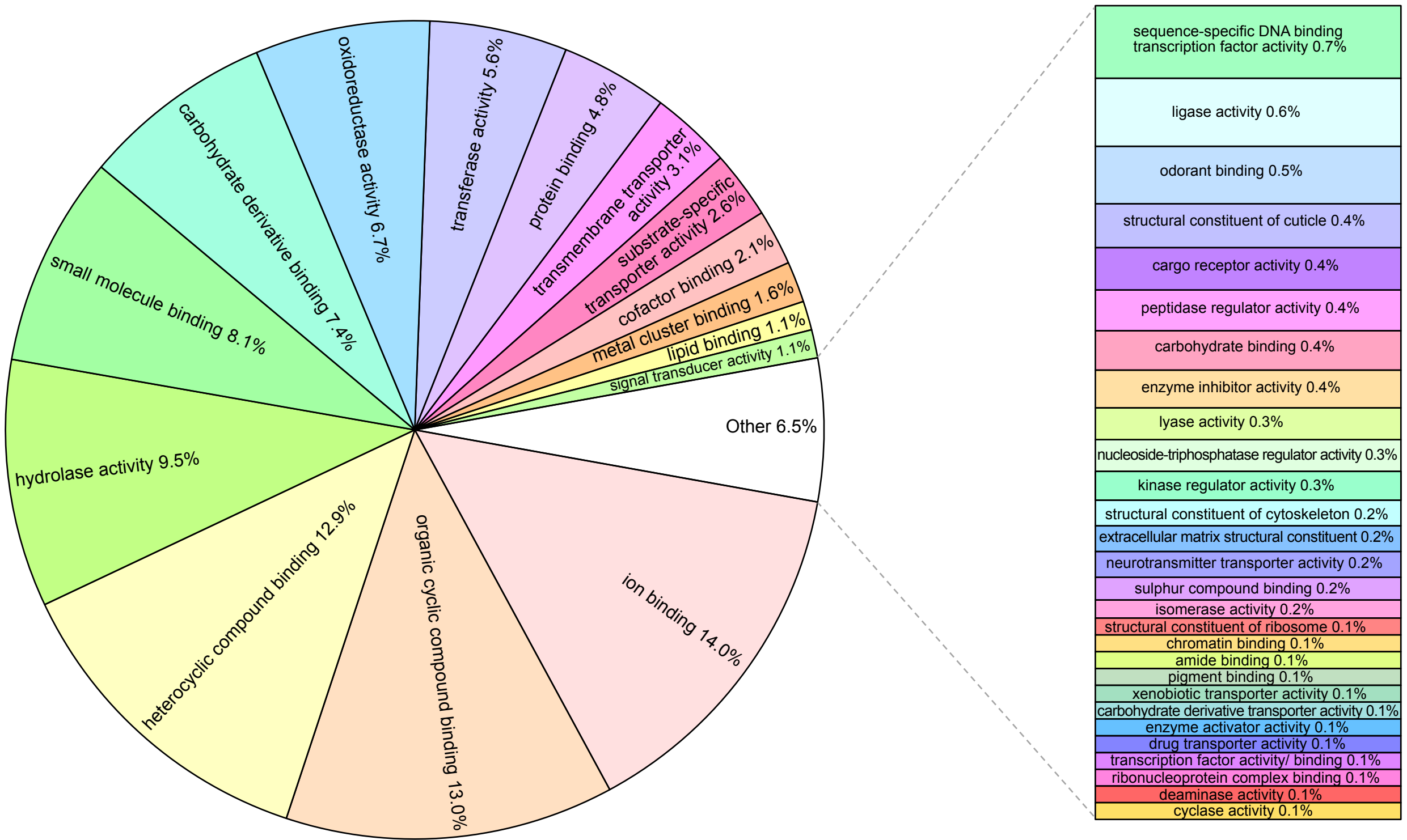

Supplement: Supplementary file 3 — Additional file 3: Figure S1. Molecular function (level 3) Gene Ontology classification of the transcripts that were differentially abundant in the different population libraries. [file 12864_2020_6956_MOESM3_ESM.pdf]

$r = 0.912$   
 $P < 0.0001$

qRT-PCR  $\text{Log}_2$  ratio

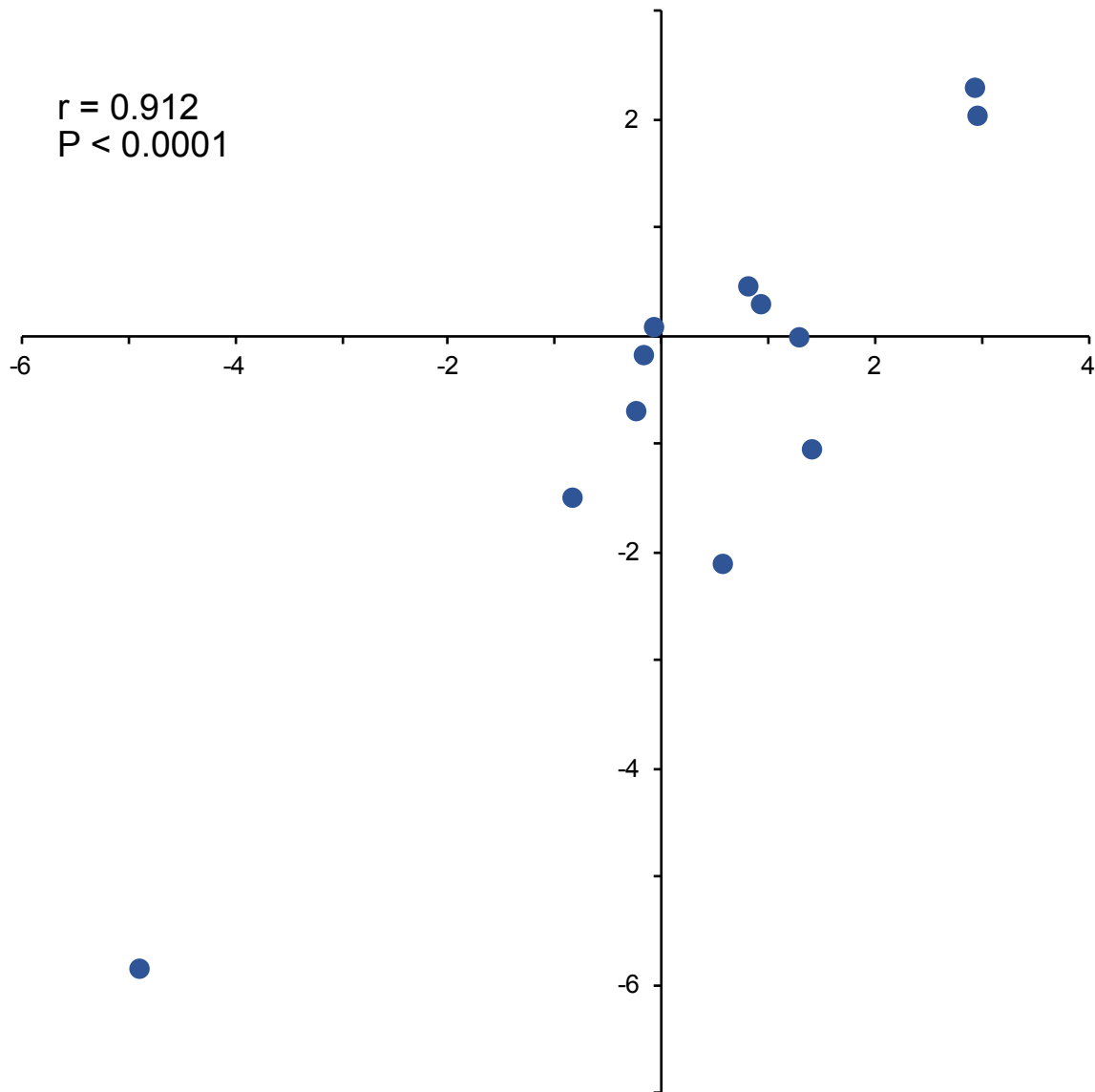

RNA-seq  $\text{Log}_2$  ratio

Supplement: Supplementary file 5 — Additional file 5: Figure S2. Validation of RNA-seq derived changes in transcript abundance with quantitative real-time RT-PCR for six genes. The log2 transformed ratios of FPKM in comparisons between population samples are plotted against the corresponding log2 transformed relative transcript abundance values obtained with real-time qRT-PCR ratios for six genes (Additional file 6: Table S9). The Pearson correlation coefficient, r = 0.912, is highly significant (P = 3.6E-05). [file 12864_2020_6956_MOESM5_ESM.pdf]

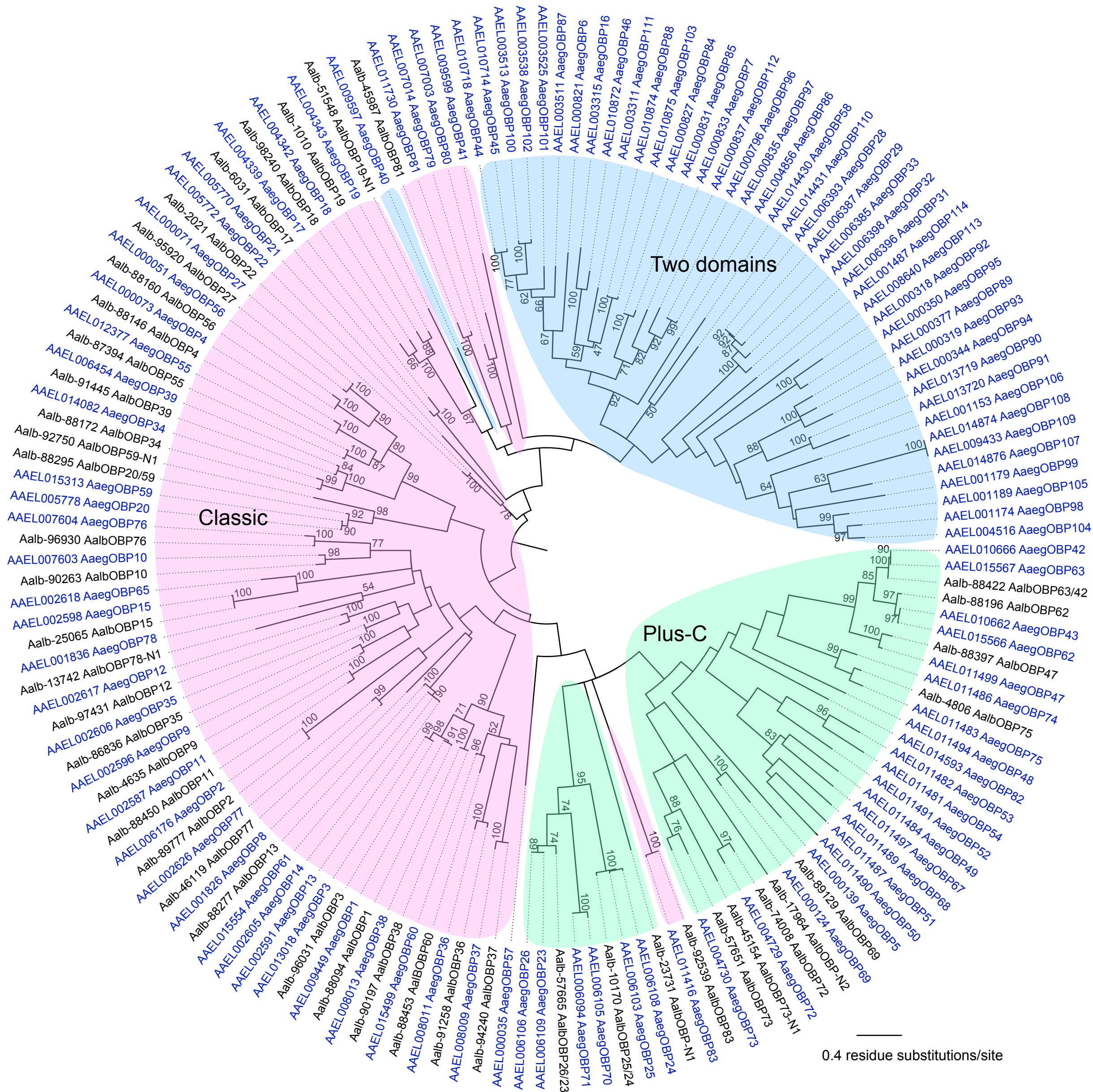

Supplement: Supplementary file 7 — Additional file 7: Figure S3. Phylogenetic relationships of OBP proteins from Ae. albopictus and Ae. aegypti. The unrooted maximum likelihood (log likelihood = − 27,485) tree was inferred using the W&G model (Whelan and Goldman 2001) with a discrete Gamma distribution. Bootstrap values greater than 50% (1000 replications) are shown. OBPs belonging to the Classic, Plus-C and Two domains subfamilies are highlighted. [file 12864_2020_6956_MOESM7_ESM.pdf]

Polymorphic sites / kilobase

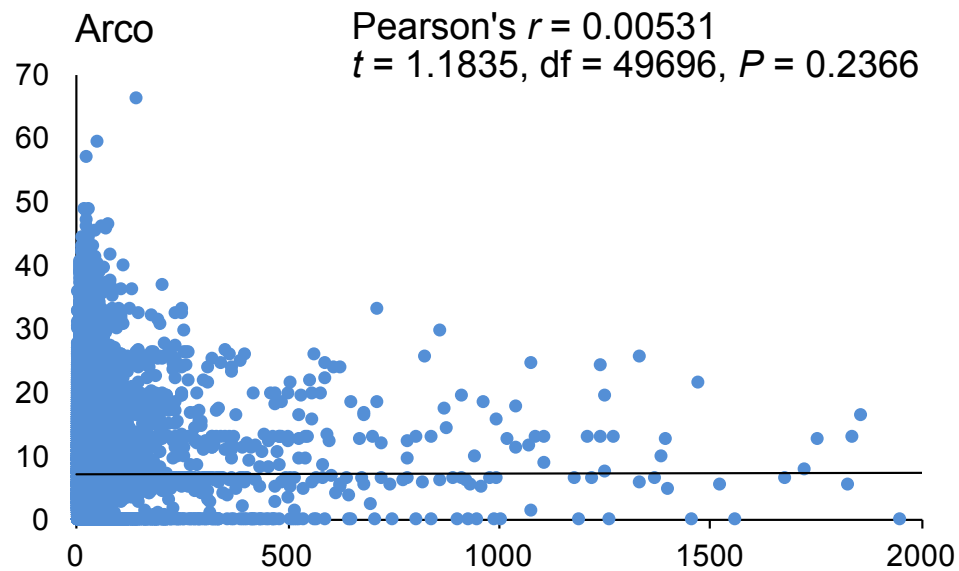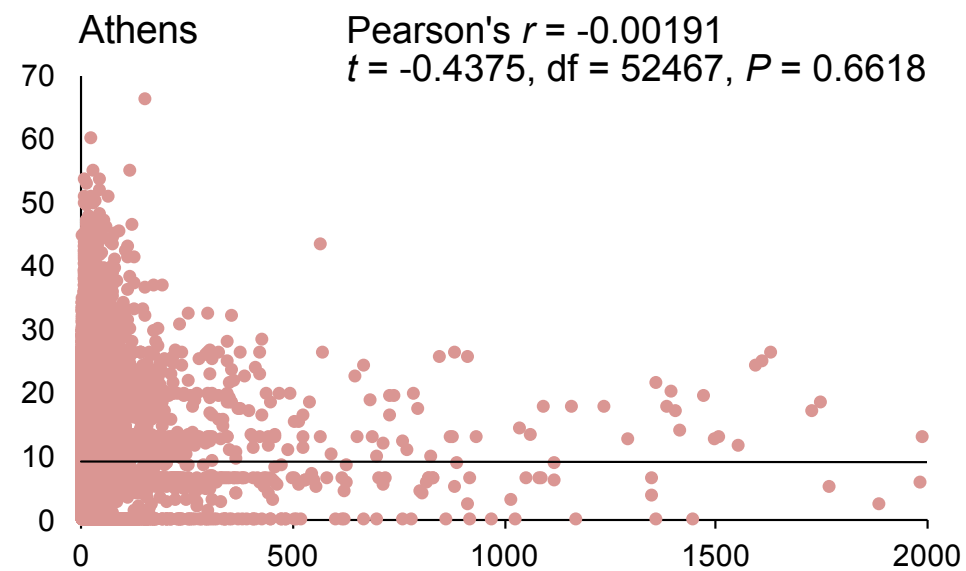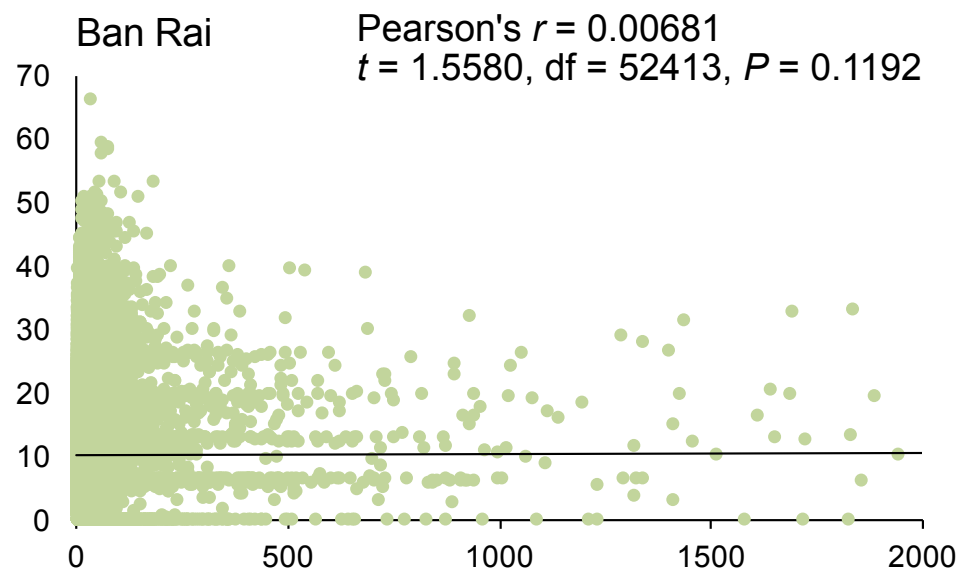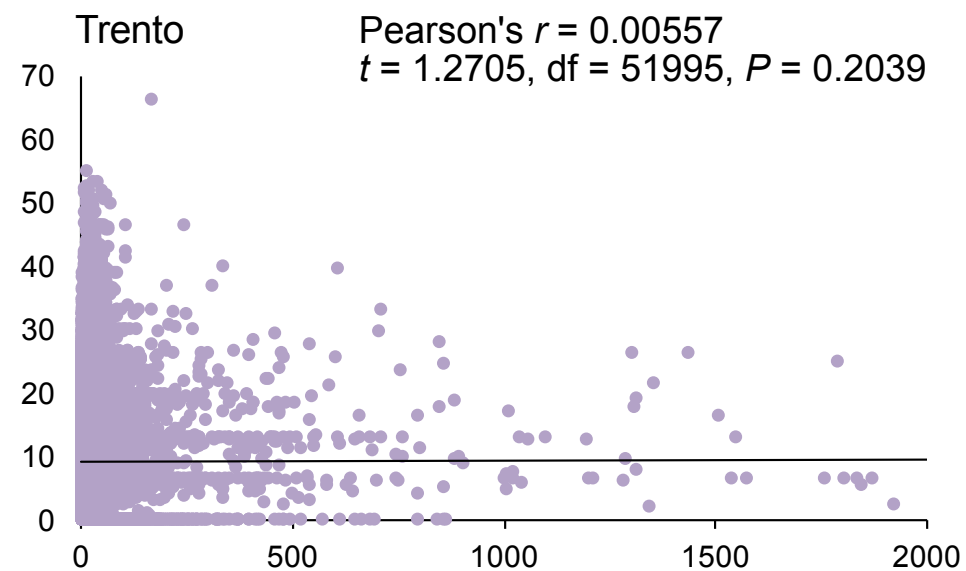

FPKM

Supplement: Supplementary file 11 — Additional file 11: Figure S5. Relationship between the number of polymorphic sites per kilobase and read depth (FPKM) for the four population samples. For clarity, the x-axes are truncated at FPKM = 2000, which precludes the vision of less than 0.09% of the data points. Trendlines are plotted on each of the graphs. [file 12864_2020_6956_MOESM11_ESM.pdf]
