# Supplementary material for: Transcriptional variation of sensory-related genes in natural populations of Aedes albopictus
Source: BMC Genomics. 2020 Aug 7;21:547. doi: 10.1186/s12864-020-06956-6 (PMC7430840; doi:10.1186/s12864-020-06956-6)
Supplement: Supplementary file 6 — Additional file 6: Table S9. Correlation of RNAseq transcript abundance ratios with real-time qRT-PCR ratios for six genes (log2 transformed). [file 12864_2020_6956_MOESM6_ESM.docx]

Table S9 Correlation of RNAseq transcript abundance ratios with real-time qRT-PCR ratios for six genes (log_2_ transformed)

|  |  | Log_2_ ratio Arco vs. Athens | | Log_2_ ratio Arco vs Ban Rai | |
| --- | --- | --- | --- | --- | --- |
| Sequence | Gene | RNA-seq | qRT-PCR | RNA-seq | qRT-PCR |
| Aalb-6031 | AalbOBP17 | 1.4190 | -1.0422 | 0.9246 | 0.2792 |
| Aalb-88196 | AalbOBP62 | 1.2950 | -0.0158 | 0.8003 | 0.4634 |
| Aalb-4806 | AalbOBP75 | 2.9583 | 2.0367 | 2.9332 | 2.2948 |
| Aalb-91878 | nompC | -0.2385 | -0.6897 | -0.8301 | -1.4989 |
| Aalb-89068 | CCEae3a | -4.8782 | -5.8477 | 0.5726 | -2.1129 |
| Aalb-87486 | cyp450 | -0.0689 | 0.0840 | -0.1569 | -0.1782 |

Pearson correlation = 0.9119, *P* = 3.6E-05
