# Supplementary material for: Transcriptional variation of sensory-related genes in natural populations of Aedes albopictus
Source: BMC Genomics. 2020 Aug 7;21:547. doi: 10.1186/s12864-020-06956-6 (PMC7430840; doi:10.1186/s12864-020-06956-6)
Supplement: Supplementary file 8 — Additional file 8: Table S10. Comparison of the odorant binding protein transcripts identified against the Lombardo (2017) transcripts. Table S11. Comparison of the odorant receptor transcripts identified against the Lombardo (2017) transcripts. [file 12864_2020_6956_MOESM8_ESM.pdf]

**Table S10 TBLASTN comparisons of OBPs identified in the transcriptomes and those identified by Lombardo et al. (2017)**

| Transcript | Name | Length (aa) | Transcript_Lombardo | Name Lombardo et al. | Length (aa) | % identity | alignment length | e-value | bit-score | Comment |
|---|---|---|---|---|---|---|---|---|---|---|
| 88094 | AalbOBP1 | 143 | AALF023075 | AalbOBP1 | 235 | 94/97 | 119 | 3.93E-80 | 235 | |
| 89777 | AalbOBP2 | 159 | Ae2SigPSigP-245707_FR2_11-195 | AalbOBP2 | 159 | 99/100 | 159 | 4.23E-115 | 322 | |
| 96031 | AalbOBP3 | 143 | Ae2SigPSigP-246894_FR3_61-234 | AalbOBP3 | 143 | 99/99 | 143 | 7.82E-104 | 292 | |
| 88146 | AalbOBP4 | 147 | Ae2SigPSigP-245019_FR5_303-456 | AalbOBP4 | 147 | 99/99 | 147 | 2.28E-96 | 273 | |
| - | - | - | AALF026669 | AalbOBP5 | 315 | - | - | - | - | |
| - | - | - | AALF017938 | AalbOBP7-N1 | 310 | - | - | - | - | |
| 4635 | AalbOBP9 | 132 | AALF024996 | AalbOBP9 | 132 | 98/99 | 132 | 1.19E-95 | 270 | |
| 90263 | AalbOBP10 | 140 | AALF027078 | AalbOBP10 | 140 | 98/99 | 140 | 1.24E-101 | 286 | |
| 88450 | AalbOBP11 | 137 | AALF007517 | AalbOBP11 | 137 | 97/98 | 137 | 7.27E-97 | 274 | |
| 97431 | AalbOBP12 | 132 | AALF019174 | AalbOBP12 | 132 | 100/100 | 132 | 1.08E-80 | 233 | |
| 88277 | AalbOBP13 | 133 | AALF019175 | AalbOBP13 | 133 | 99/99 | 133 | 1.75E-95 | 270 | |
| 25065 | AalbOBP15 | 136 | AALF019178 | AalbOBP15 | 136 | 100/100 | 136 | 1.62E-87 | 250 | |
| - | - | - | AALF018393 | AalbOBP16 | 270 | - | - | - | - | |
| 6031 | AalbOBP17 | 138 | Ae2SigPSigP-70660_FR2_3-125 | AalbOBP17 | 92 | 93/98 | 92 | 2.22E-35 | 117 | |
| 98240 | AalbOBP18 | 141 | Ae2SigPSigP-221527_FR6_753-897 | AalbOBP18 | 141 | 98/99 | 141 | 1.68E-91 | 261 | |
| 1010 | AalbOBP19 | 125 | AALF023074 | AalbOBP19 | 93 | 99/100 | 77 | 2.80E-54 | 164 | |
| 51548 | AalbOBP19-N1 | 142 | AALF025127 | - | 142 | 98/99 | 142 | 1.33E-102 | 289 | |
| 88295 | AalbOBP20/59 | 166 | AALF005057 | AalbOBP20/59 | 166 | 99/99 | 166 | 1.29E-110 | 311 | |
| 60064 | AalbOBP21 | 56 | AALF014342 | AalbOBP21 | 246 | 100/100 | 56 | 2.78E-36 | 120 | |
| 2021 | AalbOPB22 | 138 | AALF014341 | AalbOBP22 | 138 | 99/100 | 138 | 5.99E-89 | 254 | |
| 57665 | AalbOBP26/23 | 101 | AALF018601 | AalbOBP26/23 | 237 | 100/100 | 101 | 1.38E-71 | 212 | |
| 10170 | AalbOBP25/24 | 200 | AALF018602 | AalbOBP25/24 | 200 | 99/99 | 200 | 4.51E-117 | 330 | |
| 95920 | AalbOBP27 | 139 | Ae2SigPSigP-218581_FR4_17-171 | AalbOBP27 | 139 | 98/98 | 139 | 3.72E-100 | 282 | |
| - | - | - | AALF022644 | AalbOBP28 | 334 | - | - | - | - | |
| - | - | - | AALF022642 | AalbOBP33 | 636 | - | - | - | - | |
| 88172 | AalbOBP34 | 149 | AALF028521 | AalbOBP34 | 142 | 99/100 | 128 | 5.83E-80 | 232 | |
| 86836 | AalbOBP35 | 131 | AALF013191 | AalbOBP35 | 131 | 100/100 | 131 | 6.00E-79 | 228 | |
| 91258 | AalbOBP36 | 152 | AALF009587 | AalbOBP36 | 152 | 100/100 | 152 | 2.18E-115 | 322 | |
| 94240 | AalbOBP37 | 149 | Ae2SigPSigP-225473_FR6_9-169 | AalbOBP37 | 149 | 98/99 | 149 | 7.65E-99 | 280 | |
| 90197 | AalbOBP38 | 140 | Ae2SigPSigP-224714_FR1_155-257 | AalbOBP38 | 80 | 100/100 | 80 | 8.37E-56 | 168 | |
| 91445 | AalbOBP39 | 146 | AALF005090 | AalbOBP39 | 183 | 98/98 | 127 | 1.38E-80 | 235 | |
| - | - | - | AALF008889 | AalbOBP40 | 291 | - | - | - | - | |
| 88422 | AalbOBP63/42 | 157 | Ae2SigPSigP-217188_FR2_96-312 | AalbOBP63/42 | 193 | 100/100 | 157 | 9.78E-116 | 325 | |
| - | - | - | AALF015687 | AalbOBP44 | 278 | - | - | - | - | |
| - | - | - | AALF013004 | AalbOBP46 | 298 | - | - | - | - | |
| 88397 | AalbOBP47 | 191 | Ae2SigPSigP-237436_FR3_27-219 | AalbOBP47 | 191 | 100/100 | 191 | 8.75E-129 | 359 | |
| - | - | - | AALF018600 | AalbOBP48 | 213 | - | - | - | - | |
| - | - | - | AALF020568 | AalbOBP49 | 354 | - | - | - | - | |
| - | - | - | AALF020569 | AalbOBP51 | 196 | - | - | - | - | |
| - | - | - | AALF020570 | AalbOBP54 | 185 | - | - | - | - | |
| 87394 | AalbOBP55 | 151 | AALF021480 | AalbOBP55 | 151 | 100/100 | 151 | 3.99E-110 | 308 | |

| | | | | | | | | | |
|---|---|---|---|---|---|---|---|---|---|
| 88160 | AalbOBP56 | 140 | AALF026945 | AalbOBP56 | 147 | 100/100 | 130 | 4.22E-96 | 273 |
| - | - | - | AALF021771 | AalbOBP57 | 133 | - | - | - | - |
| - | - | - | AALF021668 | AalbOBP58 | 273 | - | - | - | - |
| 92750 | AalbOBP59-N1 | 155 | AALF013539 | - | 129 | 97/98 | 116 | 2.17E-65 | 195 |
| 88453 | AalbOBP60 | 122 | AALF002302 | AalbOBP60 | 145 | 99/100 | 106 | 2.37E-73 | 214 |
| 88196 | AalbOBP62 | 193 | AALF001353 | AalbOBP62 | 149 | 100/100 | 75 | 8.27E-51 | 160 |
| - | - | - | AALF019177 | AalbOBP65 | 122 | - | - | - | - |
| 89129 | AalbOBP69 | 181 | Ae2SigPSigP-30328_FR1_5-140 | AalbOBP69 | 127 | 97/99 | 127 | 4.89E-89 | 256 |
| - | - | - | AALF020565 | AalbOBP70 | 213 | - | - | - | - |
| 74008 | AalbOBP72 | 156 | AALF013104 | AalbOBP72 | 179 | 100/100 | 155 | 1.87E-113 | 318 |
| 57651 | AalbOBP73 | 166 | Ae2SigPSigP-31227_FR4_1-172 | AalbOBP73 | 168 | 99/100 | 166 | 5.75E-124 | 345 |
| 45154 | AalbOBP73-N1 | 169 | - | - | - | - | - | - | - |
| 78299 | AalbOBP74 | 90 | Ae2SigPSigP-29640_FR2_1-211 | AalbOBP74 | 192 | 99/99 | 90 | 1.09E-61 | 185 |
| 4806 | AalbOBP75 | 193 | AALF020567 | AalbOBP75 | 193 | 96/99 | 193 | 7.58E-128 | 357 |
| 96930 | AalbOBP76 | 133 | Ae2SigPSigP-244899_FR6_5-155 | AalbOBP76 | 133 | 98/100 | 133 | 1.10E-96 | 273 |
| 46119 | AalbOBP77 | 120 | AALF024997 | AalbOBP77 | 151 | 98/99 | 119 | 3.50E-85 | 244 |
| 13742 | AalbOBP78-N1 | 116 | AALF008468 | - | 136 | 98/99 | 116 | 1.32E-84 | 242 |
| - | - | - | AALF008469 | AalbOBP78 | 135 | - | - | - | - |
| - | - | - | AALF022738 | AalbOBP79 | 154 | - | - | - | - |
| - | - | - | AALF015707 | AalbOBP80 | 152 | - | - | - | - |
| 45987 | AalbOBP81 | 151 | AALF007787 | AalbOBP81 | 151 | 96/99 | 151 | 1.13E-110 | 310 |
| 92539 | AalbOBP83 | 306 | AALF018890 | AalbOBP83 | 306 | 98/99 | 306 | 2.51E-128 | 367 |
| - | - | - | AALF006399 | AalbOBP89 | 353 | - | - | - | - |
| - | - | - | AALF008134 | AalbOBP90 | 201 | - | - | - | - |
| - | - | - | AALF017937 | AalbOBP97 | 307 | - | - | - | - |
| - | - | - | AALF013138 | AalbOBP98 | 310 | - | - | - | - |
| - | - | - | AALF017970 | AalbOBP100 | 285 | - | - | - | - |
| - | - | - | AALF010900 | AalbOBP101 | 293 | - | - | - | - |
| - | - | - | AALF011068 | AalbOBP102 | 301 | - | - | - | - |
| - | - | - | AALF013003 | AalbOBP103 | 296 | - | - | - | - |
| - | - | - | AALF013136 | AalbOBP104 | 316 | - | - | - | - |
| - | - | - | AALF009317 | AalbOBP105 | 305 | - | - | - | - |
| - | - | - | AALF013134 | AalbOBP106 | 306 | - | - | - | - |
| - | - | - | AALF014081 | AalbOBP107 | 306 | - | - | - | - |
| - | - | - | AALF014080 | AalbOBP108 | 278 | - | - | - | - |
| - | - | - | AALF021669 | AalbOBP110 | 268 | - | - | - | - |
| - | - | - | AALF003679 | AalbOBP111 | 338 | - | - | - | - |
| - | - | - | AALF003184 | AalbOBP111-N1 | 291 | - | - | - | - |
| - | - | - | AALF017939 | AalbOBP112 | 299 | - | - | - | - |
| - | - | - | AALF000521 | AalbOBP113 | 273 | - | - | - | - |
| 23731 | AalbOBP-N1 | 197 | Ae2SigPSigP-20809_FR1_15-226 | - | 197 | 100/100 | 197 | 2.86E-133 | 371 |
| 17964 | AalbOBP-N2 | 103 | - | - | - | - | - | - | - |

**Table S11 TBLASTN comparisons of Ors identified in the transcriptomes and those identified by Lombardo et al. (2017)**

| Transcript | Name | Length (aa) | Transcript_Lombardo | Name Lombardo et al. | Length (aa) | % identity | alignment length | e-value | bit-score | Comment |
|---|---|---|---|---|---|---|---|---|---|---|
| 16280 | AalbOR2 | 376 | AALF011758 | AalbOr2 | 395 | 95/95 | 395 | 0 | 753 | |
| 5943 | AalbOR4 | 406 | Ae2-239510 | AalbOr4/5 | 239 | 97/99 | 239 | 3.80E-174 | 485 | |
| 82460 | AalbOR6 | 401 | AALF017217 | AalbOr6 | 275 | 99/99 | 275 | 0 | 540 | |
| 88204 | AalbOR7/ORCO | 479 | AALF000221 | AalbOrco | 464 | 99/100 | 464 | 0 | 902 | |
| 4707 | AalbOR8 | 793 | AALF006802 | AalbOr8 | 1488 | 98/99 | 801 | 0 | 1440 | |
| - | | | AALF007388 | AalbOr9 | 347 | - | - | - | - | |
| - | - | | AALF004497 | AalbOr9a | 583 | - | - | - | - | |
| 8503 | AalbOR10 | 375 | AALF007898 | AalbOr10 | 375 | 99/99 | 375 | 0 | 703 | |
| 95352 | AalbOR11 | 420 | Ae2-240598 | AalbOr11 | 420 | 99/100 | 420 | 0 | 847 | |
| 83056 | AalbOR13 | 389 | AALF017037 | AalbOr13 | 389 | 99/99 | 389 | 0 | 766 | |
| 9257 | AalbOR15 | 390 | AALF010978 | AalbOr15 | 403 | 99/100 | 390 | 0 | 797 | |
| 92214 | AalbOR19 | 400 | AALF013368 | AalbOr18/19 | 251 | 95/97 | 154 | 5.29E-103 | 305 | |
| 84246 | AalbOR20 | 396 | - | - | - | - | - | - | - | |
| 47088 | AalbOR21 | 396 | - | - | - | - | - | - | - | |
| 17244 | AalbOR22/N1 | 385 | AALF001943 | AalbOr-N1 | 390 | 92/93 | 385 | 0 | 719 | Identical except for an internal 33aa stretch |
| 5704 | AalbOR23 | 402 | AALF018383 | AalbOr23 | 349 | 99/99 | 335 | 0 | 660 | |
| 16397 | AalbOR24 | 395 | AALF021484 | AalbOr24 | 263 | 99/99 | 263 | 0 | 509 | |
| 19286 | AalbOR24-N1 | 180 | - | - | - | - | - | - | - | |
| 84055 | AalbOR25 | 381 | - | - | - | - | - | - | - | |
| 85326 | AalbOR26 | 401 | Ae2-246982 | AalbOr26 | 260 | 99/100 | 260 | 0 | 544 | |
| 80773 | AalbOR27 | 139 | AALF019852 | AalbOr27 | 330 | 100/100 | 139 | 1.09E-100 | 291 | |
| 88595 | AalbOR29 | 384 | Ae2-261366 | ND | 383 | 99/99 | 383 | 0 | 712 | |
| 17821 | AalbOR30 | 384 | AALF020411 | AalbOr30 | 384 | 83/92 | 384 | 0 | 632 | |
| 84635 | AalbOR31 | 403 | Ae2-241221 | AalbOr31 | 353 | 98/99 | 332 | 0 | 662 | |
| 81229 | AalbOR33 | 395 | - | - | - | - | - | - | - | |
| - | - | - | AALF000550 | AalbOr34 | 388 | - | - | - | - | |
| - | - | - | AALF000551 | AalbOr37 | 286 | - | - | - | - | |
| 4286 | AalbOR39 | 406 | AALF019769 | AalbOr39 | 457 | 98/99 | 393 | 0 | 804 | |
| 10769 | AalbOR42 | 391 | Ae2SigPSigP-212053_FR3_1-374 | AalbOr42 | 314 | 99/99 | 314 | 0 | 637 | |
| 80037 | AalbOR42-N1 | 386 | - | - | | - | - | - | - | |
| 15665 | AalbOR44 | 385 | Ae2SigPSigP-640_FR2_104-223 | ND | ? | 100 | 100 | 3.00E-49 | 160 | |
| 5267 | AalbOR45 | 381 | - | - | - | - | - | - | - | |
| 18518 | AalbOR45-N1 | 207 | AALF002781 | ND | 262 | 100/100 | 111 | 1.05E-61 | 192 | |
| - | - | | AALF013594 | AalbOr46 | 384 | - | - | - | - | - |
| - | - | ND | AALF007975 | AalbOr47 | - | - | - | - | - | Sequence not available in Lombardo et al. |
| - | - | - | AALF019854 | AalbOr47-N1 | 114 | - | - | - | - | |
| 97870 | AalbOR47-N2 | 383 | Ae2-241229 | ND | 287 | 100/100 | 286 | 0 | 574 | |
| 56511 | AalbOR47-N3 | 170 | - | - | - | - | - | - | - | |
| - | - | - | AALF015303 | AalbOr48 | 397 | - | - | - | - | |
| - | - | - | AALF025037 | AalbOr49 | 401 | - | - | - | - | |
| - | - | - | AALF007974 | AalbOr50 | - | - | - | - | - | |

| | | | | | | | | | | |
|---|---|---|---|---|---|---|---|---|---|---|
| 7041 | AalbOR50-N1 | 257 | AALF019855 | AalbOr50-N1 | 365 | 94/96 | 161 | 1.00E-107 | 316 | |
| 5118 | AalbOR52 | 421 | Ae2SigPSigP-256023_FR6_86-173 | AalbOr52 | 82 | 100/100 | 82 | 8.00E-56 | 178 | |
| 70366 | AalbOR55 | 395 | Ae2-89582 | AalbOr55 | 199 | 99/100 | 199 | 2.45E-149 | 420 | |
| - | - | - | AALF012995 | AalbOr58 | 398 | - | - | - | - | |
| 1114 | AalbOR59 | 250 | AALF000962 | AalbOr59 | 415 | 98/99 | 250 | 0 | 514 | |
| - | - | - | AALF003917 | AalbOr60 | 418 | - | - | - | - | |
| - | - | - | AALF018937 | AalbOr61 | 254 | - | - | - | - | |
| - | - | - | AALF018936 | AalbOr61-N1 | 186 | - | - | - | - | |
| - | - | - | AALF003919 | AalbOr61-N2 | 421 | - | - | - | - | |
| - | - | - | AALF003920 | AalbOr61-N3 | 376 | - | - | - | - | |
| 69237 | AalbOR62 | 375 | AALF000278 | AalbOr62 | 345 | 96/98 | 345 | 0 | 678 | |
| 4058 | AalbOR63 | 286 | AALF012784 | AalbOr63 | 356 | 100/100 | 246 | 1.78E-163 | 457 | |
| - | - | ND | AALF026720 | AalbOr63-N1 | - | - | - | - | - | Sequence not available in Lombardo et al. |
| 89176 | AalbOR63-N2 | 404 | Ae2SigPSigP-261786_FR6_1-222 | ND | 210 | 98/99 | 210 | 1.29E-147 | 416 | |
| 696 | AalbOR66 | 389 | - | - | - | - | - | - | - | |
| 45285 | AalbOR67 | 60 | AALF014651 | AalbOr67 | 383 | 100/100 | 60 | 6.21E-38 | 128 | |
| 47052 | AalbOR69 | 385 | AALF019062 | AalbOr69 | 342 | 89/89 | 385 | 0 | 673 | 100% identical apart from 44 aa deletion in AALF019062 |
| 73112 | AalbOR70 | 199 | Ae2SigPSigP-43314_FR6_155-284 | AalbOR70 | 110 | 100/100 | 99 | 2.66E-45 | 145 | |
| 10463 | AalbOR70-N1 | 385 | AALF019061 | AalbOr70-N1 | 320 | 100/100 | 254 | 0 | 524 | |
| 81174 | AalbOR70-N2 | 385 | - | - | - | - | - | - | - | |
| 13202 | AalbOR71 | 422 | AALF008906 | AalbOr71 | 377 | 89/89 | 422 | 0 | 687 | AALF008906 contains 45aa deletion - otherwise identical |
| 6552 | AalbOR72 | 412 | AALF004843 | AalbOr72 | 413 | 93/96 | 412 | 0 | 794 | |
| 5361 | AalbOR72-N1 | 358 | Ae2-230246 | AalbOr72-N1 | 233 | 91/96 | 233 | 1.96E-160 | 448 | |
| - | - | - | AALF012994 | AalbOr74 | 401 | - | - | - | - | |
| 69694 | AalbOR75 | 96 | AALF000084 | AalbOr75 | 703 | 100/100 | 96 | 5.32E-62 | 199 | |
| 6097 | AalbOR76 | 225 | AALF019149 | AalbOr76 | 381 | 96/99 | 225 | 3.32E-161 | 450 | |
| - | - | - | AALF000088 | AalbOr78 | 400 | - | - | - | - | |
| 9669 | AalbOR79 | 395 | AALF019151 | AalbOr79 | 395 | 98/99 | 395 | 0 | 803 | |
| 3076 | AalbOR80 | 422 | Ae2-239710 | AalbOr80 | 273 | 99/99 | 273 | 0 | 538 | |
| 97462 | AalbOR81 | 423 | Ae2SigPSigP-1103_FR4_35-441 | AalbOr81 | 350 | 97/98 | 350 | 0 | 667 | |
| - | - | - | AALF005181 | AalbOr81-N1 | 204 | - | - | - | - | |
| - | - | - | AALF027084 | AalbOr81-N2 | 401 | - | - | - | - | |
| 18076 | AalbOR83c | 353 | AALF012785 | AalbOr83c | 401 | 98/99 | 353 | 0 | 666 | |
| 83353 | AalbOR84 | 410 | Ae2SigPSigP-208879_FR6_150-233 | AalbOr84 | 74 | 100/100 | 74 | 3.76E-34 | 121 | |
| 89654 | AalbOR85 | 416 | AALF019254 | AalbOr85 | 416 | 99/99 | 416 | 0 | 778 | |
| 89563 | AalbOR87 | 424 | - | - | - | - | - | - | - | |
| 445 | AalbOR87-N1 | 353 | - | - | - | - | - | - | - | |
| 89562 | AalbOR87-N2 | 288 | - | - | - | - | - | - | - | |
| 92688 | AalbOR88 | 408 | Ae2-216959 | AalbOr88 | 308 | 99/99 | 308 | 0 | 591 | |
| 47016 | AalbOR91 | 102 | Ae2SigPSigP-221634_FR2_1-400 | AalbOr91 | 362 | 99/100 | 102 | 3.23E-69 | 210 | |
| - | - | - | AALF010815 | AalbOr92a | 387 | - | - | - | - | |
| 84524 | AalbOR94 | 123 | Ae2SigPSigP-73945_FR4_1-319 | AalbOr94 | 281 | 98/99 | 123 | 2.64E-85 | 249 | |
| 53071 | AalbOR94-N1 | 172 | - | - | - | - | - | - | - | |
| - | - | - | Ae2SigPSigP-93163_FR5_1-272 | AalbOr95 | 261 | - | - | - | - | |
| 56211 | AalbOR97 | 101 | Ae2-5507 | AalbOr97 | 391 | 97/97 | 101 | 2.04E-63 | 196 | |
| 50344 | AalbOR99 | 283 | AALF017530 | AalbOr99 | 382 | 99/99 | 283 | 1.33E-176 | 491 | |

| | | | | | | | | | |
|---|---|---|---|---|---|---|---|---|---|
| 96847 | AalbOR100 | 415 | Ae2SigPSigP-40415_FR4_1-122 | ND | 86 | 97/97 | 86 | 1.00E-51 | 167 |
| 26945 | AalbOR101 | 210 | - | - | - | - | - | - | - |
| 9171 | AalbOR102 | 363 | - | - | - | - | - | - | - |
| | | | Ae2-229490 | AalbOr104 | 234 | | | | |
| 96630 | AalbOR104-N1 | 405 | Ae2-229489 | ND | 249 | 98/99 | 249 | 0.00E+00 | 506 |
| 17596 | AalbOR109 | 150 | - | - | - | - | - | - | - |
| 47815 | AalbOR110 | 244 | - | - | - | - | - | - | - |
| 97176 | AalbOR111 | 421 | AALF016854 | AalbOr111 | 379 | 98/99 | 372 | 0 | 766 |
| 97175 | AalbOR111-N1 | 420 | Ae2-259585 | AalbOr111-N1 | 337 | 99/99 | 337 | 0 | 698 |
| 82645 | AalbOR113 | 418 | AALF027387 | AalbOr113 | 375 | 99/99 | 375 | 0 | 770 |
| - | - | - | AALF027386 | AalbOr113-N1 | 729 | - | - | - | - |
| 84373 | AalbOR113-N2 | 157 | AALF027388 | AalbOr113-N2 | 463 | 92/97 | 157 | 4.88E-103 | 302 |
| 5235 | AalbOR115 | 230 | Ae2-171283 | AalbOr115 | 295 | 96/97 | 119 | 1.14E-79 | 240 |
| - | - | - | AALF010412 | AalbOr117 | 327 | - | - | - | - |
| 45911 | AalbOR117-N1 | 169 | AALF010302 | AalbOr117-N1 | 321 | 99/100 | 167 | 6.42E-123 | 348 |
| 15754 | AalbOR117-N2 | 415 | - | - | - | - | - | - | - |
| 7651 | AalbOR117-N3 | 160 | - | - | - | - | - | - | - |
| 56571 | AalbOR117-N4 | 417 | - | - | - | - | - | - | - |
| 44012 | - | 70 | Ae2-15544 | AalbOr119 | 357 | 93/94 | 70 | 3.00E-36 | 124 |
| 53133 | AalbOR121 | 421 | - | - | - | - | - | - | - |
| 16771 | AalbOR122 | 406 | - | - | - | - | - | - | - |
| 71045 | AalbOR123 | 376 | - | - | - | - | - | - | - |
| 84576 | AalbOR125 | 310 | AALF008015 | AalbOr125 | 382 | 98/99 | 310 | 0 | 639 |
| - | - | - | AALF012996 | AalbOr-N2 | 377 | - | - | - | - |
| 41443 | AalbOR-N3 | 58 | AALF014266 | AalbOr-N3 | 354 | 100/100 | 58 | 4.49E-34 | 117 |
| - | - | - | AALF004018 | AalbOr-N4 | 372 | - | - | - | - |
| 86662 | AalbOR-N5 | 416 | AALF000712 | ND | 416 | 96/98 | 416 | 0 | 798 |
| 401 | AalbOR-N6 | 413 | - | - | - | - | - | - | - |
| 3853 | AalbOR-N7 | 414 | Ae2SigPSigP-261748_FR3_290-425 | ND | 98 | 100/100 | 98 | 2.61E-66 | 206 |
