# Supplementary material for: Transcriptional variation of sensory-related genes in natural populations of Aedes albopictus
Source: BMC Genomics. 2020 Aug 7;21:547. doi: 10.1186/s12864-020-06956-6 (PMC7430840; doi:10.1186/s12864-020-06956-6)
Supplement: Supplementary file 10 — Additional file 10: Table S12.Aedes albopictus putative odorant receptor (GR) transcripts (BLASTP against NR database). [file 12864_2020_6956_MOESM10_ESM.docx]

**Table S12.** *Aedes albopictus* putative odorant receptor (GR) transcripts (BLASTP against NR database)

|  |  | BLASTP against nr | |  |  | *Ae. aegypti* orthologue |  |  | Conserved Domains (CDD) | | |
| --- | --- | --- | --- | --- | --- | --- | --- | --- | --- | --- | --- |
| Transcript | aa | Accession | Species | e-value | % I/S | Gene | name |  | Domain | e-value | Incomplete^2^ |
| Aalb-23742 | 66 | XP_019538310.1 | *Ae. albopictus* | 2e-37 | 100/100 | AAEL002380 | GR1 |  | - | - | - |
| Aalb-54256 | 66 | XP_019550717.1 | *Ae. albopictus* | 7e-35 | 97/97 | AAEL011073 | GR19 |  | 7tm_7 | 1.64E-12 | N |
| Aalb-52731 | 69 | XP_019525826.1 | *Ae. albopictus* | 5e-37 | 99/99 | AAEL017415 | GR35 |  | - | - | - |
| Aalb-54525 | 63 | XP_019525826.1 | *Ae. albopictus* | 1e-32 | 100/100 | AAEL017415 | GR35 |  | 7tm_7 | 1.06E-06 | N |
| Aalb-45985 | 53 | XP_019527430.1 | *Ae. albopictus* | 2e-27 | 98/100 | AAEL017169 | GR36 |  | - | - | - |
| Aalb-48795 | 129 | XP_019551754.1 | *Ae. albopictus* | 2e-81 | 96/96 | AAEL017569 | GR44 |  | 7tm_7 superfamily | 4.90E-12 | N |
| Aalb-54409 | 370 | XP_019545704.1 | *Ae. albopictus* | 0.0 | 99/99 | AAEL017230^1^ | GR16^1^ |  | 7tm_7 | 6.35E-19 | - |

^1^Best hit against Vectorbase *Ae. aegypti* protein database, *e* = 2e-07, 22% identity.

^2^ N, C: incomplete at N- and/or C-terminus.
