# Supplementary material for: Transcriptional variation of sensory-related genes in natural populations of Aedes albopictus
Source: BMC Genomics. 2020 Aug 7;21:547. doi: 10.1186/s12864-020-06956-6 (PMC7430840; doi:10.1186/s12864-020-06956-6)
Supplement: Supplementary file 12 — Additional file 12: Table S13. Frequencies of synonymous and non-synonymous SNP variants in the OBP transcripts. [file 12864_2020_6956_MOESM12_ESM.docx]

Table S13. Frequencies of synonymous and non-synonymous SNPs in the OBP transcripts

| OBP transcript | OBP name | Number of SNP loci* | Number of synonymous SNP variants* | Number of non-synonymous SNP variants* |
| --- | --- | --- | --- | --- |
| Aalb-88094 | AalbOBP1 | 5 (0) | 5 (0) | 0 (0) |
| Aalb-89777 | AalbOBP2 | 6 (1) | 5 (0) | 1 (1) |
| Aalb-96031 | AalbOBP3 | 4 (0) | 4 (0) | 0 (0) |
| Aalb-88146 | AalbOBP4 | 6 (1) | 5 (0) | 1 (1) |
| Aalb-4635 | AalbOBP9 | 10 (2) | 8 (1) | 2 (1) |
| Aalb-90263 | AalbOBP10 | 10 (4) | 7 (2) | 3 (2) |
| Aalb-88450 | AalbOBP11 | 7 (1) | 5 (0) | 2 (1) |
| Aalb-97431 | AalbOBP12 | 6 (0) | 5 (0) | 1 (0) |
| Aalb-88277 | AalbOBP13 | 8 (1) | 7 (0) | 1 (1) |
| Aalb-25065 | AalbOBP15 | 5 (1) | 4 (1) | 1 (0) |
| Aalb-6031 | AalbOBP17 | 15 (2) | 11 (1) | 4 (1) |
| Aalb-98240 | AalbOBP18 | 16 (1) | 12 (0) | 4 (1) |
| Aalb-1010 | AalbOBP19 | 12 (0) | 10 (0) | 2 (0) |
| Aalb-51548 | AalbOBP19-N1 | 10 (0) | 8 (0) | 2 (0) |
| Aalb-88295 | AalbOBP20/59 | 10 (1) | 8 (0) | 2 (1) |
| Aalb-2021 | AalbOBP22 | 14 (3) | 11 (1) | 3 (2) |
| Aalb-57665 | AalbOBP26/23 | 8 (0) | 8 (0) | 0 (0) |
| Aalb-10170 | AalbOBP25/24 | 20 (2) | 19 (2) | 1 (0) |
| Aalb-95920 | AalbOBP27 | 10 (5) | 8 (3) | 2 (2) |
| Aalb-88172 | AalbOBP34 | 10 (0) | 11 (0) | 0 (0) |
| Aalb-86836 | AalbOBP35 | 5 (1) | 4 (0) | 1 (1) |
| Aalb-91258 | AalbOBP36 | 6 (2) | 2 (0) | 4 (2) |
| Aalb-94240 | AalbOBP37 | 9 (1) | 7 (0) | 2 (1) |
| Aalb-90197 | AalbOBP38 | 10 (0) | 10 (0) | 0 (0) |
| Aalb-91445 | AalbOBP39 | 10 (1) | 8 (0) | 2 (1) |
| Aalb-88422 | AalbOBP63/42 | 8 (0) | 8 (0) | 0 (0) |
| Aalb-88397 | AalbOBP47 | 18 (4) | 10 (1) | 8 (3) |
| Aalb-87394 | AalbOBP55 | 8 (0) | 8 (0) | 0 (0) |
| Aalb-88160 | AalbOBP56 | 9 (0) | 9 (0) | 0 (0) |
| Aalb-92750 | AalbOBP59-N1 | 11 (1) | 9 (1) | 3 (0) |
| Aalb-88453 | AalbOBP60 | 6 (0) | 6 (0) | 1 (0) |
| Aalb-88196 | AalbOBP62 | 7 (1) | 6 (1) | 1 (0) |
| Aalb-89129 | AalbOBP69 | 19 (2) | 18 (1) | 2 (1) |
| Aalb-74008 | AalbOBP72 | 15 (0) | 15 (0) | 0 (0) |
| Aalb-57651 | AalbOBP73 | 14 (0) | 13 (0) | 1 (0) |
| Aalb-45154 | AalbOBP73-N1 | 3 (0) | 1 (0) | 2 (0) |
| Aalb-4806 | AalbOBP75 | 19 (1) | 16 (1) | 3 (0) |
| Aalb-96930 | AalbOBP76 | 12 (3) | 12 (4)^†^ | 1 (0) |
| Aalb-46119 | AalbOBP77 | 9 (0) | 7 (0) | 2 (0) |
| Aalb-13742 | AalbOBP78-N1 | 19 (0) | 11 (0) | 8 (0) |
| Aalb-45987 | AalbOBP81 | 10 (2) | 6 (1) | 4 (1) |
| Aalb-92539 | AalbOBP83 | 20 (1) | 18 (1) | 2 (0) |
| Aalb-23731 | AalbOBP-N1 | 13 (3) | 12 (2) | 1 (1) |
| Aalb-17964 | AalbOBP-N2 | 11 (0) | 6 (0) | 5 (0) |
| Total |  | 463 | 383 (24) | 85 (25) |

* numbers in parentheses indicate SNPs in the Signal Peptide region

^†^ triallelic SNP present in Signal Peptide region
