# Supplementary material for: Transcriptional variation of sensory-related genes in natural populations of Aedes albopictus
Source: BMC Genomics. 2020 Aug 7;21:547. doi: 10.1186/s12864-020-06956-6 (PMC7430840; doi:10.1186/s12864-020-06956-6)
Supplement: Supplementary file 13 — Additional file 13: Table S14. Frequencies of synonymous and non-synonymous SNP variants in the OR transcripts. [file 12864_2020_6956_MOESM13_ESM.docx]

Table S14. Frequencies of synonymous and non-synonymous SNP variants in the OR transcripts.

|  |  |  | No. variants | No. private synonymous variants | | | | No. variants | No. private non-synonymous variants | | | |
| --- | --- | --- | --- | --- | --- | --- | --- | --- | --- | --- | --- | --- |
| OR transcript | OR name | SNP loci | synonymous | BanRai | Athens | Arco | Trento | non-synonymous | BanRai | Athens | Arco | Trento |
| Aalb-16280 | AalbOR2 | 16 | 16 |  |  |  |  | 0 |  |  |  |  |
| Aalb-5943 | AalbOR4/5 | 37 | 27 | 2 |  | 1 |  | 10 | 2 |  |  |  |
| Aalb-82460 | AalbOR6 | 19 | 17 |  |  |  |  | 2 |  | 1 |  |  |
| Aalb-88204 | AalbOR7/ORCO | 23 | 23 |  |  |  |  | 1 |  |  |  |  |
| Aalb-8503 | AalbOR10 | 24 | 23 | 1 | 1 |  |  | 1 |  |  |  |  |
| Aalb-95352 | AalbOR11 | 27 | 27 | 3 |  |  |  | 2 |  |  |  |  |
| Aalb-83056 | AalbOR13 | 19 | 12 |  |  |  |  | 7 | 1 |  |  |  |
| Aalb-9257 | AalbOR15 | 38 | 38 |  |  |  | 1 | 0 |  |  |  |  |
| Aalb-92214 | AalbOR18/19 | 26 | 12 |  |  |  |  | 14 |  |  |  |  |
| Aalb-84246 | AalbOR20 | 16 | 15 |  |  |  |  | 1 |  |  |  |  |
| Aalb-47088 | AalbOR21 | 11 | 8 |  |  |  |  | 3 |  |  |  |  |
| Aalb-17244 | AalbOR22/N1 | 28 | 17 | 1 |  |  |  | 11 | 2 |  |  | 1 |
| Aalb-5704 | AalbOR23 | 36 | 34 | 1 | 1 |  |  | 2 |  |  |  |  |
| Aalb-16397 | AalbOR24 | 11 | 7 | 1 | 1 |  |  | 4 | 2 |  |  |  |
| Aalb-19286 | AalbOR24-N1 | 11 | 8 |  |  |  |  | 4 |  |  |  | 1 |
| Aalb-84055 | AalbOR25 | 20 | 17 |  |  |  | 1 | 4 |  |  |  |  |
| Aalb-85326 | AalbOR26 | 33 | 26 |  | 1 |  |  | 7 |  |  |  |  |
| Aalb-88595 | AalbOR29 | 19 | 10 |  |  |  |  | 9 |  | 1 |  |  |
| Aalb-17821 | AalbOR30 | 7 | 5 | 1 |  |  |  | 2 |  |  |  |  |
| Aalb-84635 | AalbOR31 | 24 | 17 |  |  | 1 |  | 7 | 1 |  | 1 |  |
| Aalb-81229 | AalbOR33 | 20 | 19 |  | 1 |  |  | 1 |  |  |  |  |
| Aalb-4286 | AalbOR39 | 28 | 26 | 2 | 1 |  |  | 2 |  |  |  |  |
| Aalb-10769 | AalbOR42 | 27 | 19 | 2 |  |  |  | 9 |  |  |  |  |
| Aalb-80037 | AalbOR42-N1 | 25 | 20 |  |  |  |  | 5 |  |  |  |  |
| Aalb-15665 | AalbOR44 | 17 | 18 | 1 | 1 |  |  | 0 |  |  |  |  |
| Aalb-5267 | AalbOR45 | 36 | 29 | 2 |  |  |  | 7 |  |  |  |  |
| Aalb-18518 | AalbOR45-N1 | 8 | 0 |  |  |  |  | 8 |  |  | 1 | 1 |
| Aalb-97870 | AalbOR47-N2 | 19 | 12 | 2 |  |  |  | 7 | 1 |  |  |  |
| Aalb-56511 | AalbOR47-N3 | 5 | 3 |  |  |  |  | 2 |  |  |  |  |
| Aalb-7041 | AalbOR50-N1 | 31 | 26 | 1 |  |  |  | 5 |  |  |  |  |
| Aalb-5118 | AalbOR52 | 33 | 30 |  |  |  |  | 3 |  |  |  |  |
| Aalb-70366 | AalbOR55 | 27 | 18 |  |  |  | 3 | 5 |  | 1 |  | 1 |
| Aalb-1114 | AalbOR59 | 27 | 17 |  |  | 1 |  | 10 |  | 1 |  |  |
| Aalb-69237 | AalbOR62 | 24 | 21 | 9 |  |  | 1 | 3 | 1 |  | 1 |  |
| Aalb-4058 | AalbOR63 | 18 | 19 | 2 |  | 2 |  | 0 |  |  |  |  |
| Aalb-89176 | AalbOR63-N2 | 40 | 39 | 5 |  |  | 1 | 2 |  |  |  |  |
| Aalb-696 | AalbOR66 | 23 | 21 |  |  |  |  | 2 |  | 1 |  |  |
| Aalb-47052 | AalbOR69 | 19 | 14 | 2 |  |  |  | 5 |  |  |  |  |
| Aalb-73112 | AalbOR70 | 18 | 13 |  | 1 |  |  | 5 | 1 | 1 |  |  |
| Aalb-10463 | AalbOR70-N1 | 19 | 14 | 3 |  |  |  | 5 |  |  |  |  |
| Aalb-81174 | AalbOR70-N2 | 32 | 28 | 2 | 3 |  |  | 5 |  |  |  |  |
| Aalb-13202 | AalbOR71 | 32 | 28 | 1 | 3 |  | 2 | 5 |  | 3 |  |  |
| Aalb-6552 | AalbOR72 | 21 | 12 | 2 |  |  |  | 9 |  | 3 |  |  |
| Aalb-6097 | AalbOR76 | 23 | 19 | 1 | 1 |  |  | 4 |  |  |  |  |
| Aalb-9669 | AalbOR79 | 38 | 25 |  |  |  |  | 13 |  | 1 | 1 |  |
| Aalb-3076 | AalbOR80 | 17 | 10 |  |  |  | 1 | 7 | 1 | 1 |  |  |
| Aalb-97462 | AalbOR81 | 28 | 12 | 5 |  |  |  | 16 |  | 1 |  |  |
| Aalb-18076 | AalbOR83c | 23 | 17 | 1 | 2 |  |  | 6 | 2 |  |  | 1 |
| Aalb-83353 | AalbOR84 | 27 | 17 |  |  |  |  | 10 | 1 |  |  |  |
| Aalb-89654 | AalbOR85 | 19 | 14 |  |  |  |  | 5 |  |  |  |  |
| Aalb-89563 | AalbOR87 | 32 | 23 | 2 | 2 |  | 1 | 9 |  |  |  |  |
| Aalb-445 | AalbOR87-N1 | 22 | 18 | 1 |  | 1 | 1 | 4 |  |  |  |  |
| Aalb-89562 | AalbOR87-N2 | 21 | 12 |  | 2 |  | 1 | 9 | 1 |  | 1 |  |
| Aalb-92688 | AalbOR88 | 31 | 30 | 1 | 1 |  | 1 | 3 |  | 1 |  |  |
| Aalb-53071 | AalbOR94-N1 | 12 | 2 |  |  |  |  | 10 |  |  |  | 1 |
| Aalb-50344 | AalbOR99 | 19 | 17 |  |  |  |  | 2 |  |  | 1 |  |
| Aalb-96847 | AalbOR100 | 31 | 18 | 1 | 1 |  |  | 13 | 2 |  |  |  |
| Aalb-26945 | AalbOR101 | 22 | 14 |  | 3 |  | 1 | 9 | 2 | 1 |  |  |
| Aalb-9171 | AalbOR102 | 34 | 29 | 4 | 3 |  | 1 | 5 | 1 | 1 |  |  |
| Aalb-96630 | AalbOR104-N1 | 41 | 22 | 2 |  |  |  | 19 | 1 | 1 |  |  |
| Aalb-17596 | AalbOR109 | 11 | 10 | 1 |  |  |  | 2 |  | 1 |  |  |
| Aalb-47815 | AalbOR110 | 19 | 9 | 1 |  |  |  | 10 |  | 1 |  | 1 |
| Aalb-97176 | AalbOR111 | 35 | 26 |  |  |  |  | 11 |  |  |  |  |
| Aalb-97175 | AalbOR111-N1 | 34 | 24 |  |  |  |  | 10 |  | 1 |  |  |
| Aalb-82645 | AalbOR113 | 38 | 33 | 2 | 5 |  |  | 5 |  | 1 |  | 1 |
| Aalb-84373 | AalbOR113-N2 | 16 | 10 | 1 | 1 |  |  | 6 |  |  | 1 |  |
| Aalb-5235 | AalbOR115 | 28 | 22 | 1 | 5 | 1 |  | 6 | 2 |  |  |  |
| Aalb-45911 | AalbOR117-N1 | 9 | 6 |  |  |  |  | 3 |  |  |  |  |
| Aalb-15754 | AalbOR117-N2 | 30 | 19 | 3 | 1 |  |  | 11 |  |  |  |  |
| Aalb-7651 | AalbOR117-N3 | 16 | 10 |  | 2 |  |  | 6 | 2 |  |  |  |
| Aalb-56571 | AalbOR117-N4 | 22 | 7 |  | 1 |  | 1 | 15 | 2 | 1 |  |  |
| Aalb-53133 | AalbOR121 | 20 | 12 | 2 |  |  |  | 8 |  |  |  |  |
| Aalb-16771 | AalbOR122 | 28 | 19 | 5 |  | 2 |  | 9 | 3 | 1 |  |  |
| Aalb-71045 | AalbOR123 | 29 | 20 | 2 | 1 | 1 | 1 | 9 |  |  |  | 1 |
| Aalb-84576 | AalbOR125 | 28 | 24 | 1 |  |  |  | 4 |  |  |  |  |
| Aalb-86662 | AalbOR-N5 | 25 | 16 | 3 |  |  |  | 9 |  | 1 |  |  |
| Aalb-401 | AalbOR-N6 | 42 | 27 | 1 | 2 |  |  | 15 | 2 |  |  | 1 |
| Aalb-3853 | AalbOR-N7 | 36 | 31 |  |  |  |  | 5 |  |  |  |  |
| Total |  | 1900 | 1410 | 87 | 47 | 10 | 18 | 484 | 33 | 26 | 7 | 10 |
