# Supplementary material for: Transcriptional variation of sensory-related genes in natural populations of Aedes albopictus
Source: BMC Genomics. 2020 Aug 7;21:547. doi: 10.1186/s12864-020-06956-6 (PMC7430840; doi:10.1186/s12864-020-06956-6)
Supplement: Supplementary file 9 — Additional file 9: Figure S4. Phylogenetic relationships of OR proteins from Ae. albopictus and Ae. aegypti. The unrooted maximum likelihood (log likelihood = − 84,078) tree was inferred using the JTT model (Jones et al. 1992) with a discrete Gamma distribution and amino acid frequencies (JTT + G + F). Bootstrap values greater than 50% (1000 replications) are shown. [file 12864_2020_6956_MOESM9_ESM.pdf]

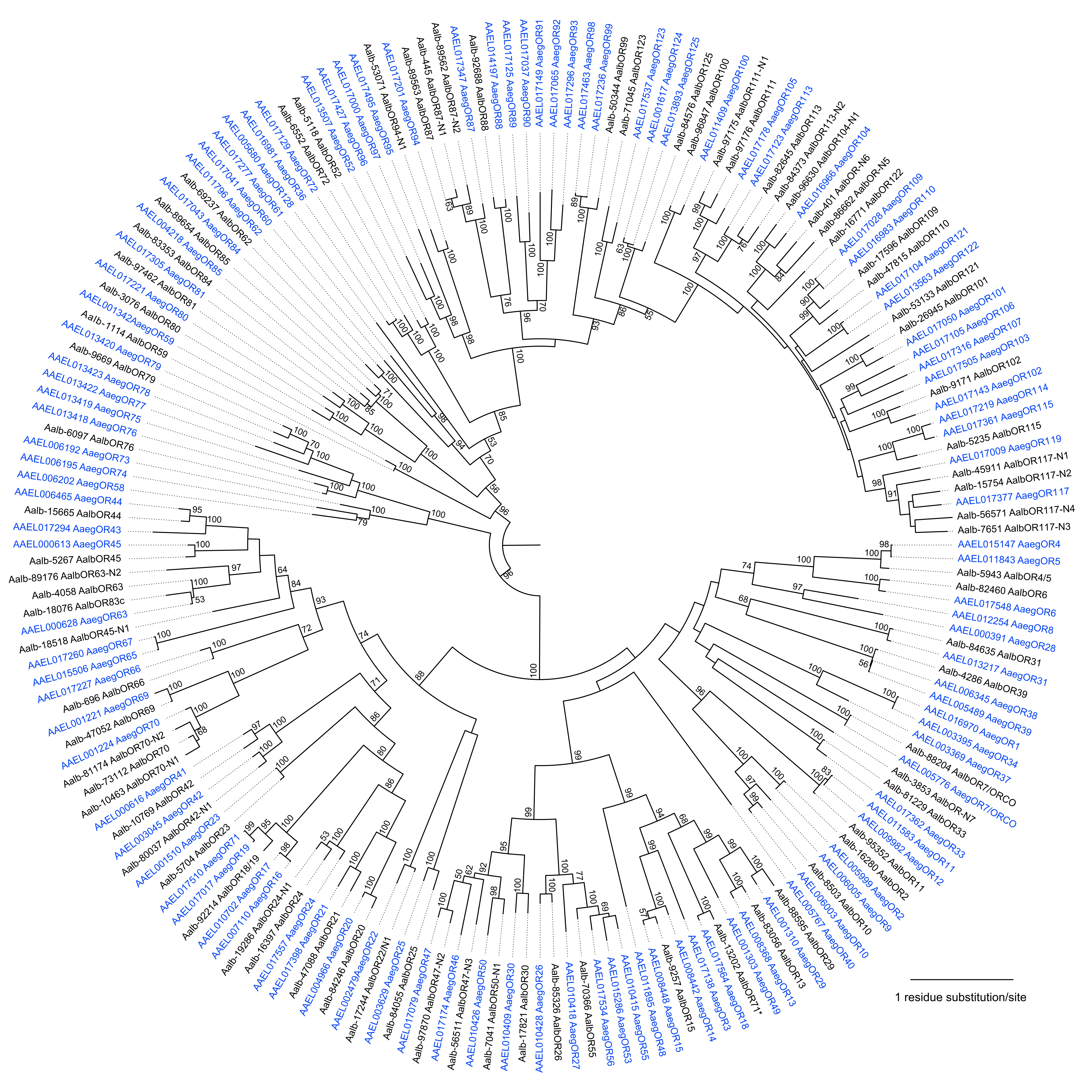

AAEL015147 AaegOR4
AAEL011843 AaegOR5
Aalb-5943 AalbOR4/5
Aalb-82460 AalbOR6
AAEL017548 AaegOR6
AAEL012254 AaegOR8
AAEL000391 AaegOR28
Aalb-84635 AalbOR31
AAEL013217 AaegOR31
Aalb-4286 AalbOR39
AAEL006345 AaegOR38
AAEL005489 AaegOR39
AAEL016970 AaegOR1
AAEL003395 AaegOR34
AAEL003369 AaegOR37
Aalb-88204 AalbOR7/ORCO
AAEL005776 AaegOR7/ORCO
Aalb-3853 AalbOR-N7
Aalb-81229 AalbOR33
AAEL017362 AaegOR33
AAEL011583 AaegOR11
AAEL009982 AaegOR12
Aalb-95352 AalbOR11
Aalb-16280 AalbOR2
AAEL005999 AaegOR2
AAEL006005 AaegOR9
Aalb-8503 AalbOR10
AAEL006003 AaegOR10
AAEL005767 AaegOR40
Aalb-88595 AalbOR29
AAEL001310 AaegOR29
Aalb-83056 AalbOR13
AAEL008368 AaegOR13
AAEL001303 AaegOR49
Aalb-13202 AalbOR71*
AAEL017564 AaegOR18
AAEL017138 AaegOR3
AAEL008442 AaegOR14
Aalb-9257 AalbOR15
AAEL008448 AaegOR15
AAEL011895 AaegOR48
AAEL010415 AaegOR55
AAEL015286 AaegOR53
AAEL017534 AaegOR56
Aalb-70366 AalbOR55
AAEL010418 AaegOR27
Aalb-85326 AalbOR26
AAEL010428 AaegOR26
Aalb-17821 AalbOR30
AAEL010409 AaegOR30
Aalb-7041 AalbOR50-N1
AAEL010426 AaegOR50
Aalb-56511 AalbOR47-N3
AAEL017174 AaegOR46
Aalb-97870 AalbOR47-N2
AAEL017079 AaegOR47
Aalb-84055 AalbOR25
AAEL003629 AaegOR25
Aalb-17244 AalbOR22/N1
AAEL002479AaegOR22
Aalb-84246 AalbOR20
AAEL004966 AaegOR20
Aalb-47088 AalbOR21
AAEL017398 AaegOR21
AAEL017557 AaegOR24
Aalb-16397 AalbOR24
Aalb-19286 AalbOR24-N1
AAEL007110 AaegOR16
AAEL010702 AaegOR17
Aalb-92214 AalbOR18/19
AAEL017017 AaegOR19
AAEL017510 AaegOR71
Aalb-5704 AalbOR23
AAEL001510 AaegOR23
Aalb-80037 AalbOR42-N1
AAEL003045 AaegOR42
Aalb-10769 AalbOR42
AAEL000616 AaegOR41
Aalb-10463 AalbOR70-N1
Aalb-73112 AalbOR70
Aalb-81174 AalbOR70-N2
AAEL001224 AaegOR70
Aalb-47052 AalbOR69
AAEL001221 AaegOR69
Aalb-696 AalbOR66
AAEL017227 AaegOR66
AAEL015506 AaegOR65
AAEL017260 AaegOR67
Aalb-18518 AalbOR45-N1
AAEL000628 AaegOR63
Aalb-18076 AalbOR83c
Aalb-4058 AalbOR63
Aalb-89176 AalbOR63-N2
Aalb-5267 AalbOR45
AAEL000613 AaegOR45
AAEL017294 AaegOR43
Aalb-15665 AalbOR44
AAEL006465 AaegOR44
AAEL006202 AaegOR58
AAEL006195 AaegOR74
AAEL006192 AaegOR73
Aalb-6097 AalbOR76
AAEL013418 AaegOR76
AAEL013419 AaegOR75
AAEL013422 AaegOR77
AAEL013423 AaegOR78
Aalb-9669 AalbOR79
AAEL013420 AaegOR79
Aalb-1114 AalbOR59
AAEL001342AaegOR59
Aalb-3076 AalbOR80
AAEL017221 AaegOR80
Aalb-97462 AalbOR81
AAEL017305 AaegOR81
Aalb-83353 AalbOR84
AAEL004218 AaegOR85
Aalb-89654 AalbOR85
AAEL017043 AaegOR84
Aalb-69237 AalbOR62
AAEL011796 AaegOR62
AAEL017041 AaegOR61
AAEL017277 AaegOR60
AAEL005680 AaegOR128
AAEL016981 AaegOR36
AAEL017129 AaegOR72
Aalb-6552 AalbOR72
Aalb-5118 AalbOR52
AAEL013507 AaegOR52
AAEL017427 AaegOR96
AAEL017000 AaegOR97
AAEL017495 AaegOR95
Aalb-53071 AalbOR94-N1
AAEL017201 AaegOR94
Aalb-89563 AalbOR87
Aalb-445 AalbOR87-N1
Aalb-89562 AalbOR87-N2
AAEL017347 AaegOR87
Aalb-92688 AalbOR88
AAEL014197 AaegOR88
AAEL017125 AaegOR89
AAEL017037 AaegOR90
AAEL017149 AaegOR91
AAEL017065 AaegOR92
AAEL017296 AaegOR93
AAEL017463 AaegOR98
AAEL017236 AaegOR99
Aalb-50344 AalbOR99
Aalb-71045 AalbOR123
AAEL017537 AaegOR123
AAEL001617 AaegOR124
AAEL013893 AaegOR125
Aalb-84576 AalbOR125
Aalb-96847 AalbOR100
AAEL011409 AaegOR100
Aalb-97175 AalbOR111-N1
Aalb-97176 AalbOR111
AAEL017178 AaegOR105
AAEL017123 AaegOR113
Aalb-82645 AalbOR113-N2
Aalb-84373 AalbOR113
Aalb-96630 AalbOR104-N1
AAEL016966 AaegOR104
Aalb-401 AalbOR-N6
Aalb-86662 AalbOR-N5
Aalb-16771 AalbOR122
AAEL017028 AaegOR109
AAEL016983 AaegOR110
Aalb-17596 AalbOR109
Aalb-47815 AalbOR110
AAEL017104 AaegOR121
AAEL013563 AaegOR122
Aalb-53133 AalbOR121
Aalb-26945 AalbOR101
AAEL017050 AaegOR101
AAEL017105 AaegOR106
AAEL017316 AaegOR107
AAEL017505 AaegOR103
Aalb-9171 AalbOR102
AAEL017143 AaegOR102
AAEL017219 AaegOR114
AAEL017361 AaegOR115
Aalb-5235 AalbOR115
AAEL017009 AaegOR119
Aalb-45911 AalbOR117-N1
Aalb-15754 AalbOR117-N2
AAEL017377 AaegOR117
Aalb-56571 AalbOR117-N4
Aalb-7651 AalbOR117-N3
1 residue substitution/site
